# Base-Promoted One-Pot Synthesis of Pyridine Derivatives via Aromatic Alkyne Annulation Using Benzamides as Nitrogen Source

**DOI:** 10.3390/molecules26216599

**Published:** 2021-10-31

**Authors:** Hina Mehmood, Muhammad Asif Iqbal, Muhammad Naeem Ashiq, Ruimao Hua

**Affiliations:** 1 Key Laboratory of Organic Optoelectronics & Molecular Engineering of Ministry of Education, Department of Chemistry, Tsinghua University, Beijing 100084, China; n-he16@mails.tsinghua.edu.cn (H.M.); asif_90pk@yahoo.com (M.A.I.); 2 Institute of Chemical Sciences, Bahauddin Zakariya University, Multan 608000, Pakistan; naeembzu@bzu.edu.pk

**Keywords:** base-promoted, alkyne annulation, pyridine derivatives, benzamide as nitrogen source

## Abstract

In the presence of Cs_2_CO_3_, the first simple, efficient, and one-pot procedure for the synthesis of 3,5-diaryl pyridines via a variety of aromatic terminal alkynes with benzamides as the nitrogen source in sulfolane is described. The formation of pyridine derivatives accompanies the outcome of 1,3-diaryl propenes, which are also useful intermediates in organic synthesis. Thus, pyridine ring results from a formal [2+2+1+1] cyclocondensation of three alkynes with benzamides, and one of the alkynes provides one carbon, whilst benzamides provide a nitrogen source only. A new transformation of alkynes as well as new utility of benzamide are found in this work.

## 1. Introduction

**Scheme 1:**
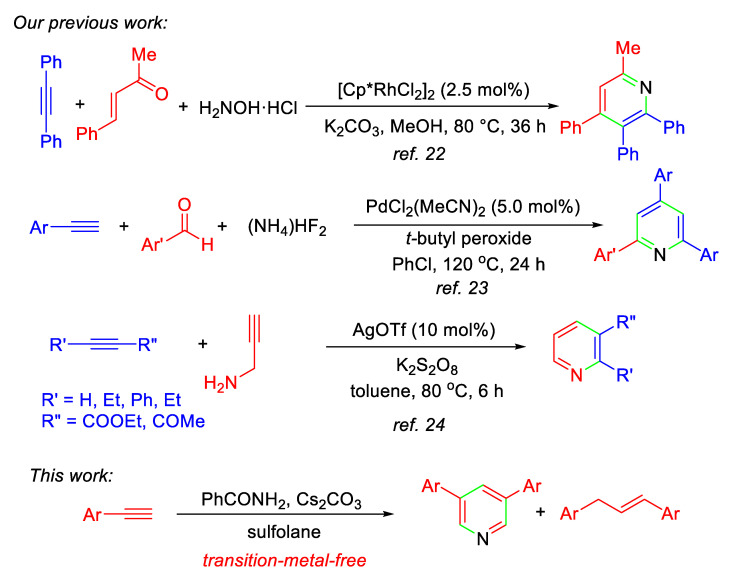
One-pot formation of pyridines by alkyne annulation reported by Hua’s group.

Pyridine derivatives are one of the most important and fundamental six-membered nitrogen-heterocyclic compounds, and pyridine ring is the core structure not only in pharmaceutical compounds [1,2,3,4], but also in natural products [5,6,7]. Therefore, development of synthetic methods for constructing pyridine ring is an interesting and enduring research topic in organic chemistry [5,8,9,10,11,12,13,14,15,16,17]. Among them, alkyne annulation with nitrogen-containing substrates has been well-documented [10,11,12,13,14,17]. We are interested in the development of alkyne annulation protocols for the synthesis of carbo- and heterocyclic compounds in a one-step procedure [18,19,20,21], as well as in the construction of pyridine ring starting from alkynes catalyzed by transition-metal complexes (Scheme 1) [22,23,24]. Encouraged by our recent success in the development of base-promoted formation of C-N and C-O bonds and their applications in the synthesis of heterocyclic compounds [25,26,27,28,29,30], in this paper we report a new protocol for the one-pot formation of 3,5-diaryl pyridines from aromatic terminal alkynes and benzamide promoted by Cs_2_CO_3_ in sulfolane, although the synthetic methods of 3,5-diaryl pyridines without use of alkyne have been recently reported [31,32,33]. In this procedure, benzamides are firstly used as the nitrogen source to provide nitrogen atom only, and the reactions also produce 1,3-diarylpropenes as the by-product.

## 2. Results and Discussion

Our initial purposes are to develop a base-promoted cyclocondensation of alkynes with benzamide to form a nitrogen-heterocyclic compound. When the mixture of phenyl acetylene (**1a**, 2.0 mmol), benzamide (**2a**, 1.0 mmol), and KO*^t^*Bu (2.0 mmol) in DMSO (2.0 mL) was heated at 135 °C for 24 h, the analyses of the reaction mixture by GC-MS disclosed the formation of 3,5-diphenylpyridine (**3aa**) and 1,3-diphenylpropene in trace amount. We are very interested in the formation of 3,5-disubstituted pyridines, since there has never been a report on the synthesis of 3,5-disubstituted pyridines in one-pot manner starting from alkynes; in addition, it is also a new transformation of alkynes. Therefore, we decided to optimize the reaction conditions to establish and provide an efficient synthetic method for access to 3,5-disubstituted pyridines by this new type of alkyne annulation protocol. On the basis of the formation of **3aa** and the structure of the by-product, it can be confirmed that **3aa** formation requires 4.0 equivalents of **1a** as shown in Scheme 1. Therefore, the optimizing reaction conditions were performed with the use of 4.0 mmol of **1a** under different conditions.

As shown in Table 1, in DMSO, the reactions of **1a** (4.0 mmol) with benzamide (**2a**, 2.0 mmol) in the presence of 4.0 mmol of KO*^t^*Bu, KOH, and K_2_CO_3_ resulted in a trace amount of **3aa** formation, confirmed by GCMS of the reaction mixtures (entries 1–3). In the case of Cs_2_CO_3_ used, delightedly, **3aa** could be isolated from the reaction mixture in 20% yield (the yield was based on the total amount of **1a** used, entry 4). The structure of **3aa** was confirmed by its NMR spectroscopic data, and the formation of the pyridine ring was unambiguously confirmed by its x-ray diffraction studies (3,5-Diphenyl pyridine (**3aa**) is a known compound, the structure was confirmed by its spectroscopic data and x-ray diffrac-tion studies. See Supplementary File and CCDC Number 2112477). The utility of other solvents, such as THF, 1,4-dioxane, DMF, and DMAc (*N*,*N*-dimethylacetamide) gave the trace amount and low yield of **3aa** (entries 5–8). In addition, sulfolane (tetramethylene sulfone, or 2,3,4,5-tetrahydrothiophen-1,1-dioxide, undried) is a highly stable and versatile dipolar aprotic laboratory and industrial solvent commonly used in organic synthesis to drastically enhance the rate and selectivity [34], and this solvent is expected to have superior solubility for alkali metal salts [35]. Thus, we repeated the reactions in sulfolane with the use of different amounts of **2a**; Cs_2_CO_3_, the desired **3aa,** was obtained in low to good yields (entries 9–12). Among them, the best reaction conditions of **2a** (1.0 mmol) and Cs_2_CO_3_ (2.5 mmol) produced **3aa** in 77% yield (based on half the amount of **1a** used) (entry 11). In this case, the by-product of 1,3-diphenyl propene, which has highly potential application and is not easily available by traditional organic synthesis, was isolated in a comparable yield (75%, based on half the amount of **1a** used). (Their ^1^H, ^13^C NMR spectroscopic data and/or GC-MS are reported in Supplementary File), accompanied with the formation of benzoic acid (confirmed by GCMS after work-up by the addition of water, it is not isolated) (see in Supplementary File). With the use of 1.0 mmol of Cs_2_CO_3_, repeating the reactions in DMSO, DMF, formamide, and DMAc again gave either a low yield of **3aa**, or no product at all (entries 13–16). If **2a** was replaced by NH_4_OAc or acetamide as the nitrogen source, no desired product was formed (entries 17–18). Acetamide does not work, which is maybe due to being more basic than benzamide [36]. In addition, no product formation was observed in the absence of a base (entry 19).

**Scheme 2:**
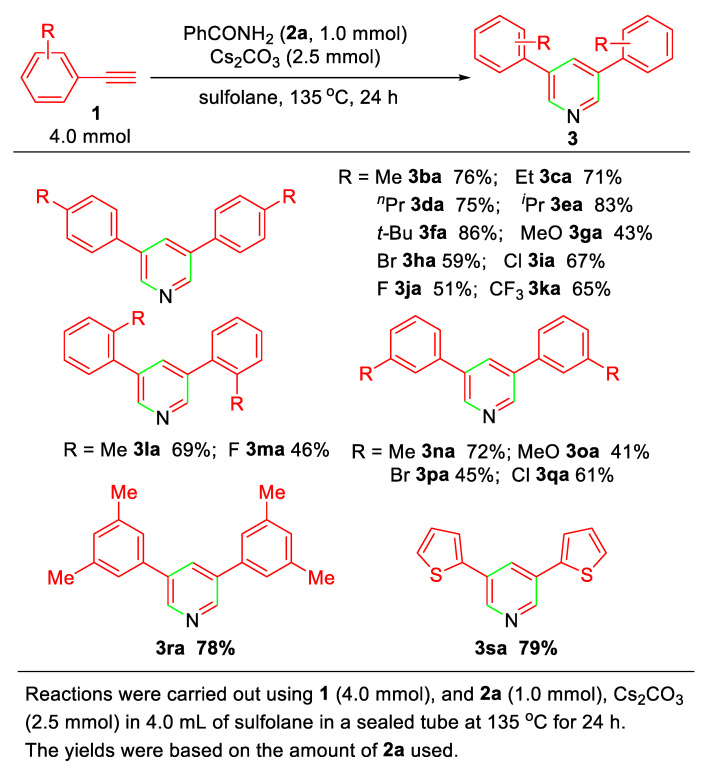
Substrate scope of pyridine synthesis.

Under the optimized reaction conditions (entry 11 of Table 1), the substrate scope of the present alkyne annulation with **2a** affording 3,5-disubstituted pyridines was then investigated. As concluded in Scheme 2, aromatic terminal alkynes bearing electron-donating (alkyl group) and electron-withdrawing substituents (Cl, F, CF_3_) could undergo the annulation to give the corresponding pyridines **3ba**~**3sa** in moderate to good yields, but an apparent dependence of the electronic effects of the substituents in aromatic terminal alkynes was observed. Thus, alkynes **1b**~**1f**, **1l**, **1n,** and **1r** bearing electron-donating groups (R = Me, Et, *^n^*Pr, *^i^*Pr, and *t*-Bu) at the *para*-, *ortho*-, or *meta*-position show the higher reactivity compared to the halogen-substituted alkynes (R = Br, Cl, F, **1h**~**1k**, **1m**, **1p**~**1q**).

The present annulation was also applicable to heteroaromatic terminal alkynes. For example, subjecting 2-ethynylthiophene (**1s**) to the standard reaction conditions afforded 3,5-di(thiophen-2-yl)pyridine (**3sa**) in 79% yield.

It should be noted that under the standard reaction conditions, two exceptions of the substituent effect were observed. The electron-rich *para*-methoxyphenylacetylenes (**1g**) and *meta*-methoxyphenylacetylenes (**1o**) show comparatively low reactivity to give the corresponding pyridines in 43% and 41% yields, respectively. For that reason, the considerable amount of enamine derivatives that resulted from the addition reaction of **1g** or **1o** with **2a** under basic conditions was detected in the reaction mixtures by GCMS, which is due to the strong electron-donating methoxy group that suppresses the formation of amide anion for further transformation (*vide infra*).

In addition, it is notable that either aliphatic terminal alkynes or internal alkynes, as well as 1-ethynyl-4-nitrobenzene used as the substrates, resulted in no formation of pyridine derivatives at all.

**Scheme 3:**
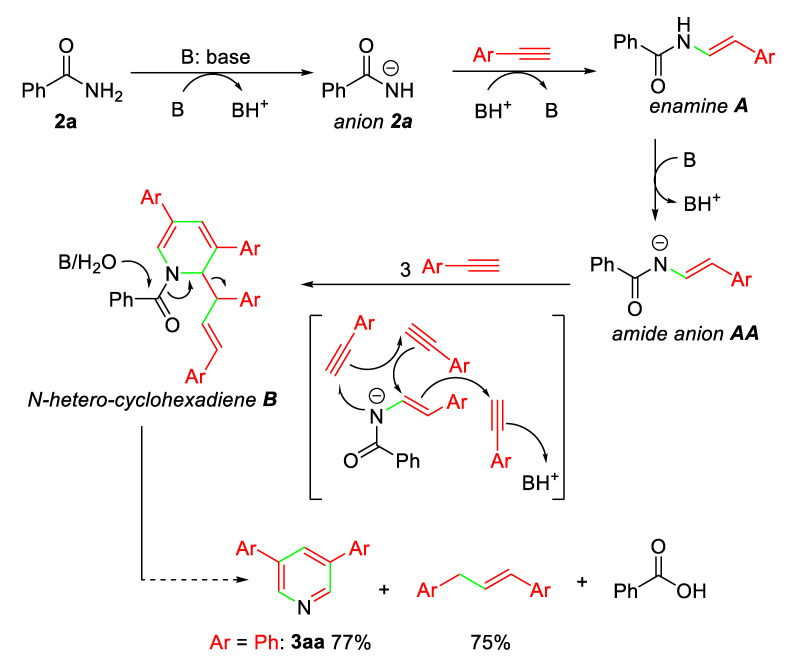
Possible mechanism for pyridine formation.

On the basis of the 3,5-diaryl pyridine and 1,3-diaryl propene formation, a proposed mechanism for the present base-promoted annulation of alkynes with benzamides to form pyridine ring is depicted in Scheme 3. It involves the common addition reaction of benzamides to terminal alkyne promoted by the base to give enamine intermediate **A**, which forms amide anion **AA** and then undergoes cycloaddition with three alkynes to afford nitrogen-heterocyclohexadiene intermediate **B**. Under basic conditions and with a small amount of water (wet solvent), intermediate **B** is considered to be converted into 3,5-diaryl pyridine and 1,3-diaryl propene via cleavage of C-N and C-C bonds.

According to the proposed mechanism, the formation of **3aa** and 1,3-diphenyl propene in the comparable yields (77% *vs* 75%) is easily understood. Also, the low reactivity of electron-rich *para*-methoxyphenylacetylenes (**1g**) and *meta*-methoxyphenylacetylenes (**1o**) is reasonably due to the strong electron-donating methoxy group that decreases the formation of amide anion **AA** (Ar = *para*-/*meta*-MeOC_6_H_4_) for further transformation.

Since acetamide is more basic than benzamide [36], when it was used as the nitrogen source, **3aa** could not form due to the difficult formation of anion **2a** (Table 1, entry 18).

**Scheme 4:**
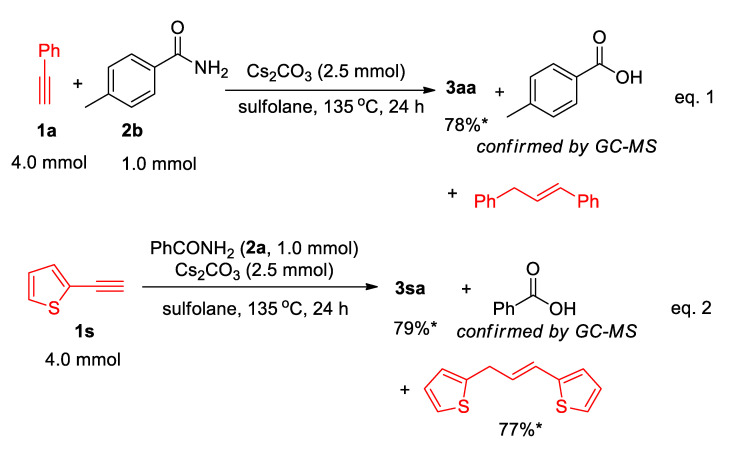
Isolation and confirmation of by-product. *: the yields were calculated on the basis of the half amount of alkyne used.

In addition, the reactions of **1a** with *para*-methylbenzamide (**2b**) gave **3aa** (78% yield), *para*-methylbenzoic acid (see in Supplementary File), and 1,3-diphenylpropene, which confirm again that the benzamide **2b** is used as the nitrogen source only (Scheme 4 (eq.1)). Moreover, in the formation of **3sa** (Scheme 2), the corresponding (*E*)-2,2′-(prop-1-ene-1,3-diyl)dithiophene was isolated in 77% yield (see in Supplementary File), and the yield is also comparable to the yield of **3sa** (79%) (Scheme 4 (eq.2)).

## 3. Materials and Methods

### 3.1. General Methods

All commercial reagents are analytically pure and used directly without further purification. Nuclear magnetic resonance (NMR) spectra were recorded on an ECA-400 spectrometer (JEOL, Tokyo, Japan) using CDCl_3_ as solvent at 298 K. ^1^H NMR (400 MHz) chemical shifts (δ) were referenced to internal standard TMS (for 1H, δ = 0.00 ppm). ^13^C NMR (100 MHz) chemical shifts were referenced to internal solvent CDCl_3_ (for ^13^C, δ = 77.16 ppm). Mass spectra (MS) were obtained on a GC-MS-QP2010S (Shimadzu, Tokyo, Japan) with a PEG-25M column, and the high-resolution mass spectra (HRMS) with electron spray ionization (ESI) were obtained with a micrOTOF-Q spectrometer (Agilent, California, CA, USA).

### 3.2. Typical Experimental Procedure for the Synthesis of 3,5-Diphenyl Pyridine (**3aa**)

A mixture of phenylacetylene (1a, 408.1 mg, 4.0 mmol), benzamide (**2a**, 121.5 mg, 1.0 mmol), and Cs_2_CO_3_ (815.1 mg, 2.5 mmol) in sulfolane (4.0 mL) in a 25 mL screw-capped thick-walled Pyrex tube was stirred at 135 °C for 24 h in an oil bath. After the reaction mixture was cooled to room temperature, it was poured into a solvent mixture of water (50.0 mL) and ethyl acetate (25.0 mL), and the two phases were then separated. The aqueous layer was extracted with ethyl acetate (3 × 15.0 mL), and the combined organic extracts were dried over anhydrous Mg_2_SO_4_. After removing the solvent under reduced pressure, the residue was purified by column chromatography on silica gel with petroleum ether/ethyl acetate (gradient mixture ratio from 100:0 to 90:10) as eluent to afford **3aa** as a white solid (177.9 mg, 77%). (*E*)-1,3-diphenylpropene was isolated in 75% yield.

The structural characterization data for all the products are reported in the Supplementary Materials.

## 4. Conclusions

In summary, the present work provides a simple and efficient method for the synthesis of 3,5-diaryl pyridines by Cs_2_CO_3_-promoted annulation of aromatic terminal alkynes with benzamides as nitrogen sources in sulfolane, along with the formation of 1,3-diaryl propenes as by-product. Noteworthy features of this procedure include the one-pot manner with a wide range of readily available alkynes under transition-metal-free conditions to afford 3,5-diaryl pyridines with high chemoselectivity, and benzamides were firstly used as nitrogen sources in the construction of pyridine. In addition, the present pyridine ring formation resulted from a formal [2+2+1+1] cyclocondensation of three alkynes and benzamides, one of the alkynes provided one carbon and benzamides provided the nitrogen atom. This is a new transformation of alkyne and shows the new utility of benzamides in organic synthesis.

## Figures and Tables

**Table 1:** Optimizing reaction conditions for 3,5-diphenylpyridine (**3aa**) formation *^a^*. 
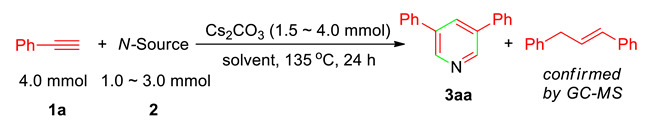

Entry	2a (mmol)	Base (mmol)	Solvent	Yield (%)
1	benzamide (2.0)	KO*^t^*Bu (4.0)	DMSO	trace
2	benzamide (2.0)	KOH (4.0)	DMSO	trace
3	benzamide (2.0)	K_2_CO_3_ (4.0)	DMSO	trace
4	benzamide (2.0)	Cs_2_CO_3_ (4.0)	DMSO	20 *^b^*
5	benzamide (2.0)	Cs_2_CO_3_ (4.0)	THF	0
6	benzamide (2.0)	Cs_2_CO_3_ (4.0)	dioxane	trace
7	benzamide (2.0)	Cs_2_CO_3_ (4.0)	DMF	trace
8	benzamide (2.0)	Cs_2_CO_3_ (4.0)	DMAc	15 *^b^*
9	benzamide (2.0)	Cs_2_CO_3_ (4.0)	sulfolane	25 *^b^*
10	benzamide (3.0)	Cs_2_CO_3_ (4.0)	sulfolane	16 *^b^*
**11**	**benzamide (1.0)**	**Cs_2_CO_3_ (2.5)**	**sulfolane**	**77 *^c^***
12	benzamide (1.0)	Cs_2_CO_3_ (1.5)	sulfolane	63 *^c^*
13	benzamide (1.0)	Cs_2_CO_3_ (2.5)	DMSO	33 *^c^*
14	benzamide (1.0)	Cs_2_CO_3_ (2.5)	DMF	0
15	benzamide (1.0)	Cs_2_CO_3_ (2.5)	formamide	0
16	benzamide (1.0)	Cs_2_CO_3_ (2.5)	DMAc	25 *^c^*
17	NH_4_OAc (1.0)	Cs_2_CO_3_ (2.5)	sulfolane	0
18	acetamide (1.0)	Cs_2_CO_3_ (2.5)	sulfolane	0
19	benzamide (1.0)	--	sulfolane	0

*^a^* Reactions were carried out using **1a** (4.0 mmol), and **2**, and base in 4.0 mL of solvent in a sealed tube at 135 °C for 24 h. *^b^* Yields of **3aa** were calculated on basis of the amount of **1a**. *^c^* Yields of **3aa** were calculated on basis of the half amount of **1a**.

## Data Availability

Not applicable.

## Supplementary Materials

The following are available online at www.mdpi.com/link. The characterization data of the known products, copies of ^1^H and ^13^C NMR charts of all products, X-ray structural details of **3aa**, and part by-product’s NMR spectroscopic data and GC-MS.

## Sample Availability

The sample of compounds are not available from authors.
