# Supplementary material for: Base-Promoted One-Pot Synthesis of Pyridine Derivatives via Aromatic Alkyne Annulation Using Benzamides as Nitrogen Source
Source: Molecules. 2021 Oct 31;26(21):6599. doi: 10.3390/molecules26216599 (PMC8587654; doi:10.3390/molecules26216599)
Supplement: Supplementary file 1 [file molecules-26-06599-s001.zip › molecules-1422919-supplementary.pdf]

Supplementary File

Base-Promoted One-Pot Synthesis of Pyridine Derivatives via Aromatic Alkyne Annulation Using Benzamides as Nitrogen Source

Hina Mehmood [1], Muhammad Asif Iqbal [1], Muhammad Naeem Ashiq [2], and Ruimao Hua [1,*]
[1] Department of Chemistry, Tsinghua University, Key Laboratory of Organic Optoelectronics & Molecular Engineering of Ministry of Education, Beijing 100084, China
[2] Institute of Chemical Sciences, Bahauddin Zakariya University, Multan 608000, Pakistan

Contents Page

1. Characterization data of products S2
2. Copies of ^{1}H NMR and ^{13}C NMR spectra of all the products S5
3. X-ray structural details of **3aa** S24
4. Part by-product's NMR spectroscopic data and GC-MS S32

1. Characterization data of known products

3,5-Diphenylpyridine **(3aa)** [1]: White solid (177.9 mg, 77%); ^{1}H NMR (400 MHz, $CDCl_3$) δ 8.83 (s, 2H), 8.05 (s, 1H), 7.65 (d, *J* = 7.3 Hz, 4H), 7.53 – 5.41 (m, 6H); ^{13}C NMR (100 MHz, $CDCl_3$) δ 147.1, 137.9, 136.7, 133.0, 129.2, 128.3, 127.4; GC-MS m/z: 231 (M^+).

3,5-Di-*p*-tolylpyridine (**3ba**) [2]: White solid (197.3 mg, 76%); ^{1}H NMR (400 MHz, $CDCl_3$) δ 8.78 (s, 2H), 8.01 (s, 1H), 7.54 (d, *J* = 8.0 Hz, 4H), 7.31 (d, *J* = 7.9 Hz, 4H); ^{13}C NMR (100 MHz, $CDCl_3$) δ 146.7, 138.7, 136.6, 135.1, 132.5, 129.9, 127.2, 21.3; GC-MS m/z: 259 (M^+).

3,5-Bis(4-ethylphenyl)pyridine **(3ca)** [3]: White solid (203.8 mg, 71%); ^{1}H NMR (400 MHz, $CDCl_3$) δ 8.77 (s, 2H), 8.02 (s, 1H), 7.55 (d, *J* = 8.1 Hz, 4H), 7.32 (d, *J* = 8.0 Hz, 4H), 2.71 (q, *J* = 7.6 Hz, 4H), 1.28 (t, *J* = 7.6 Hz, 6H); ^{13}C NMR (100 MHz, $CDCl_3$) δ 146.6, 144.6, 136.7, 135.2, 132.7, 128.7, 127.3, 28.7, 15.6; GC-MS m/z: 287 (M^+).

3,5-Bis(4-propylphenyl)pyridine (**3da**): White solid (236.6 mg, 75%); mp: 71.6 ~ 73.0 ºC; ^{1}H NMR (400 MHz, $CDCl_3$) δ 8.82 (s, 2H), 8.04 (s, 1H), 7.56 (d, J = 8.1 Hz, 4H), 7.32 (d, J = 8.0 Hz, 4H), 2.66 (t, J = 7.6, 4H), 1.77 – 1.61 (m, 4H), 0.99 (t, J = 7.3Hz, 6H); ^{13}C-NMR (100 MHz, $CDCl_3$) δ 146.6, 143.0, 135.3, 132.6, 129.3, 127.2, 37.8, 24.6, 14.0; HRMS (ESI) m/z: $[M + H]^+$ calcd for $C_{23}H_{26}N$, 316.1987; found, 316.1985.

3,5-Bis(4-isopropylphenyl)pyridine (**3ea**): White Solid (261.4 mg, 83%); mp: 83.0 ~ 84.8 ºC; ^{1}H NMR (400 MHz, $CDCl_3$) δ 8.81 (s, 2H), 8.05 (s, 1H), 7.59 (d, J = 7.7 Hz, 4H), 7.38 (d, J = 7.7 Hz, 4H), 3.07 – 2.93 (m, 2H), 1.33 (d, J = 6.9 Hz, 12H); ^{13}C NMR (100 MHz, $CDCl_3$) δ 149.1, 146.6, 136.6, 135.3, 132.7, 127.3, 127.2, 33.9, 24.0; HRMS (ESI) m/z: $[M + H]^+$ calcd for $C_{23}H_{26}N$, 316.1987; found, 316.1985.

3,5-Bis(4-(*t*-butyl)phenyl)pyridine (**3fa**): White solid (294.4 mg, 86%); mp: 100.2 ~ 101.8 ºC; ^{1}H NMR (400 MHz, $CDCl_3$) δ 8.85 (s, 2H), 8.08 (s, 1H), 7.67 – 7.51 (m, 8H), 1.42 (s, 18H); ^{13}C NMR (100 MHz, $CDCl_3$) δ 151.3, 146.7, 136.4, 134.9, 132.5, 126.9, 126.1, 34.6, 31.4; HRMS (ESI) m/z: $[M + H]^+$ calcd for $C_{25}H_{30}N$, 344.2300; found, 344.2306.

3,5-Bis(4-methoxyphenyl)pyridine (**3ga**) [1]: White Solid (125.1 mg, 43%); ^{1}H NMR (400 MHz, $CDCl_3$) δ 8.74 (s, 2H), 7.96 (s, 1H), 7.58 (d, *J* = 8.7 Hz, 4H), 7.03 (d, *J* = 8.7 Hz, 4H), 3.87 (s, 6H); ^{13}C NMR (100 MHz, $CDCl_3$) δ 159.9, 146.1, 136.4, 132.1, 130.4, 128.4, 114.7, 55.5; GC-MS m/z: 291 (M^+).

3,5-Bis(4-bromophenyl)pyridine (**3ha**) [2]: White solid (229.5 mg, 59%); ^{1}H NMR (400 MHz, $CDCl_3$) δ 8.80 (s, 2H), 7.96 (s, 1H), 7.63 (d, *J* = 8.4 Hz, 4H), 7.50 (d, *J* = 8.5 Hz, 4H); ^{13}C NMR (100 MHz, $CDCl_3$) δ 147.1, 136.5, 135.8, 132.5, 132.4, 128.9, 122.9; GC-MS m/z: 389 (M^+).

3,5-Bis(4-chlorophenyl)pyridine (**3ia**) [1]: White solid (200.3 mg, 67%); ^{1}H NMR (400 MHz, $CDCl_3$) δ 8.80 (s, 2H), 7.96 (s, 1H), 7.56 (d, *J* = 8.4 Hz, 4H), 7.47 (d, *J* = 8.4 Hz, 4H); ^{13}C NMR (100 MHz, $CDCl_3$) δ 147.1, 136.1, 135.7, 134.7, 132.6, 129.5, 128.6; GC-MS m/z: 299 (M^+).

3,5-Bis(4-fluorophenyl)pyridine (**3ja**) [1]: White Solid (136.1 mg, 51%); ^{1}H NMR (400 MHz, $CDCl_3$) δ 8.79 (s, 2H), 7.95 (s, 1H), 7.68 – 7.49 (m, 4H), 7.24 – 7.11 (m, 4H); ^{13}C NMR (100 MHz,

$CDCl_3$) δ 163.2 (d, *J* = 247.0 Hz), 146.9, 133.9, 133.8, 132.7, 129.07 (d, *J* = 8.0 Hz), 116.3 (d, *J* = 22.0 Hz); GC-MS m/z: 267 (M^+).

3,5-Bis[4-(trifluoromethyl)phenyl]pyridine (**3ka**) [2]: White solid (238.3 mg, 65%); ^1H NMR (400 MHz, $CDCl_3$) δ 8.89 (s, 2H), 8.07 (s, 1H), 7.83 – 7.73 (m, 8H); ^{13}C NMR (100 MHz, $CDCl_3$) δ 147.9, 141.0, 135.6, 133.2, 130.7 (q, *J* = 32.7 Hz), 127.7, 126.3 (q, *J* = 3.8 Hz), 124.1 (q, *J* = 272.1 Hz); GC-MS m/z: 367 (M^+).

3,5-di-*o*-Tolylpyridine (**3la**) [2]: White solid (178.6 mg, 69%); ^1H NMR (400 MHz, $CDCl_3$) δ 8.60 (s, 2H), 7.66 (s, 1H), 7.36 – 7.27 (m, 8H), 2.35 (s, 6H); ^{13}C NMR (100 MHz, $CDCl_3$) δ 148.3, 138.0, 137.1, 136.8, 135.7, 130.7, 130.0, 128.2, 126.5, 20.5; GC-MS m/z: 259 (M^+).

3,5-Bis(2-fluorophenyl)pyridine (**3ma**) [3]: White solid (122.8 mg, 46%); ^1H NMR (400 MHz, $CDCl_3$) δ 8.80 (s, 2H), 8.07 (s, 1H), 7.50 (t, *J* = 7.4 Hz, 2H), 7.45 – 7.34 (m, 2H), 7.33 – 7.16 (m, 4H); ^{13}C NMR (100 MHz, $CDCl_3$) δ 160.0 (d, *J* = 248.0 Hz), 148.7, 148.6, 136.7, 131.5, 130.73, 130.70, 130.3, 130.2, 125.6. 125.4, 124.9, 124.8, 116.5 (d, *J* = 23.0 Hz); GC-MS m/z: 267 (M^+).

3,5-Di-*m*-tolylpyridine **(3na)**[3]: White solid (186.4 mg, 72%); ^1H NMR (400 MHz, $CDCl_3$) δ 8.80 (s, 2H), 8.03 (s, 1H), 7.88 (d, *J* = 7.6 Hz, 1H), 7.48 – 7.35 (m, 6H), 7.24 (s, 1H), 2.46 (s, 6H); ^{13}C NMR (100 MHz, $CDCl_3$) δ 147.0, 138.9, 137.9, 136.8, 133.0, 129.1, 129.0, 128.1, 124.5, 21.6; GC-MS m/z: 259 (M^+).

3,5-Bis(3-methoxyphenyl)pyridine (**3oa**) [2]: White solid (119.3 mg, 41%); ^1H NMR (400 MHz, $CDCl_3$) δ 8.81 (s, 2H), 8.03 (s, 1H), 7.42 (t, *J* = 7.9 Hz, 2H), 7.26 (s, 2H), 7.22 (d, *J* = 7.7 Hz, 2H), 6.98 – 6.96 (m, 2H), 3.89 (s, 6H); ^{13}C NMR (100 MHz, $CDCl_3$) δ 160.3, 147.2, 139.3, 136.6, 133.1, 130.3, 119.8, 113.6, 113.1, 55.5; GC-MS m/z: 291 (M^+).

3,5-Bis(3-bromophenyl)pyridine (**3pa**) [1]: White solid (173.1 mg, 45%); ^1H NMR (400 MHz, $CDCl_3$) δ 8.81 (s, 2H), 7.97 (s, 1H), 7.78 (s, 2H), 7.61 – 7.51 (m, 4H), 7.38 (t, *J* = 7.9 Hz, 2H); ^{13}C-NMR (100 MHz, $CDCl_3$) δ 147.5, 139.7, 135.5, 132.9, 131.5, 130.8, 130.4, 126.0, 123.4; GC-MS m/z: 386 (M^+).

3,5-Bis(3-chlorophenyl)pyridine (**3qa**) [2]: White solid (182.3 mg, 61%); ^1H NMR (400 MHz, $CDCl_3$) δ 8.81 (s, 2H), 7.97 (s, 1H), 7.61 (s, 2H), 7.51 (d, *J* = 7.0 Hz, 2H), 7.46 – 7.36 (m, 4H); ^{13}C NMR (100 MHz, $CDCl_3$) δ 147.4, 139.3, 135.5, 135.2, 132.9, 130.5, 128.5, 127.4, 125.5; GC-MS m/z: 299 (M^+).

3,5-Bis(3,5-dimethylphenyl)pyridine (**3ra**): White solid (223.7 mg, 78%); mp 106.3 ~ 107.5 ºC; ^1H NMR (400 MHz, $CDCl_3$) δ 8.80 (s, 2H), 8.02 (s, 1H), 7.27 (s, 4H), 7.09 (s, 2H), 2.43 (s, 12H); ^{13}C NMR (100 MHz, $CDCl_3$) δ 146.9, 138.7, 137.9, 136.8, 132.9, 129.9, 125.2, 21.5; HRMS (ESI) m/z: $[M + H]^+$ calcd for $C_{21}H_{22}N$, 288.1674; found, 288.1671.

3,5-Di(thiophen-2-yl)pyridine (**3sa**) [4]: White solid (191.9 mg, 79%); ^1H NMR (400 MHz, $CDCl_3$) δ 8.76 (s, 2H), 7.99 (s, 1H), 7.46 – 7.37 (m, 4H), 7.14 – 7.12 (m, 2H); ^{13}C-NMR (100 MHz, $CDCl_3$) δ 145.6, 140.0, 130.5, 129.9, 128.4, 126.3, 124.6; GC-MS m/z: 243 (M^+).

References

[1] Sathish, M.; Chetna, J.; Krishna, N. H.; Shankaraiah, N.; Alarifi, A.; Kamal, A. Iron-mediated one-pot synthesis of 3,5-diarylpyridines from β-nitrostyrenes. *J. Org. Chem.* **2016**, *81*, 2159-2165.

[2] Ranjani, G.; Nagarajan, R. Insight into copper catalysis: In situ formed nano Cu_2O in Suzuki-Miyaura cross-coupling of aryl/indolyl boronates. *Org. Lett.* **2017**, *19*, 3974-3977.

[3] Yan, R.; Zhou, X.; Li, M.; Li, X.; Kang, X.; Liu, X.; Huo, X.; Huang, G. Metal-free synthesis of substituted pyridines from aldehydes and NH_4OAc under air. *RSC Adv.* **2014**, *4*, 50369-50372.

[4] Johnson, S. N.; Ellington, T. L.; Ngo, D, T.; Nevarez, J. L.; Sparks, N.; Rheingold, A, L.; Watkins, D. L.; Tschumper, G. S. Probing non-covalent interactions driving molecular assembly in organo-electronic building blocks. *CrystEngComm* **2019**, *21*, 3151-3157.

2. Copies of ^{1}H NMR and ^{13}C NMR spectra of all the products

^{1}H NMR spectrum of compound **3aa** (400 MHz, $CDCl_3$)

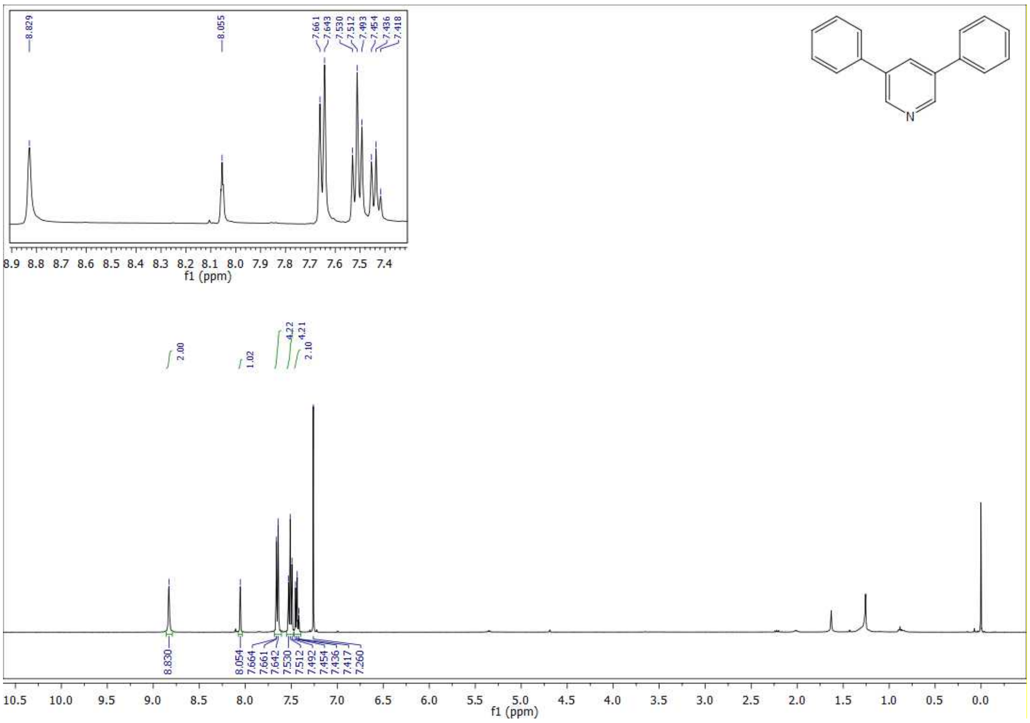

^{13}C NMR spectrum of compound **3aa** (100 MHz, $CDCl_3$)

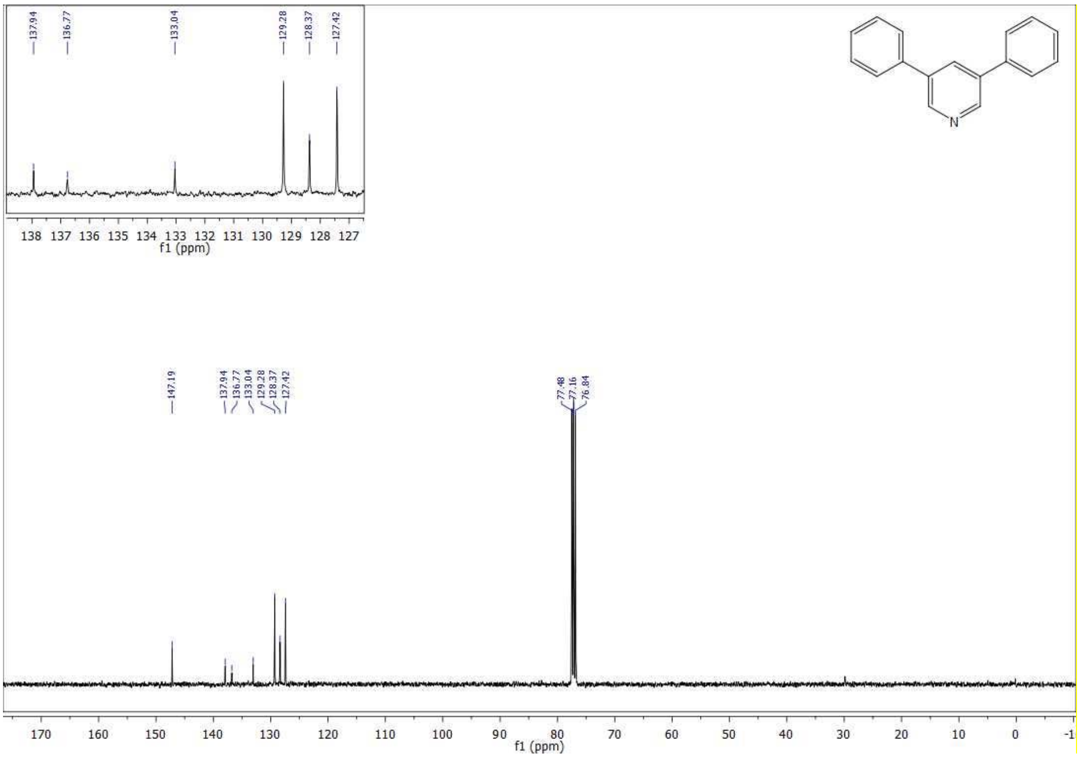

^{1}H NMR spectrum of compound **3ba** (400 MHz, $CDCl_3$)

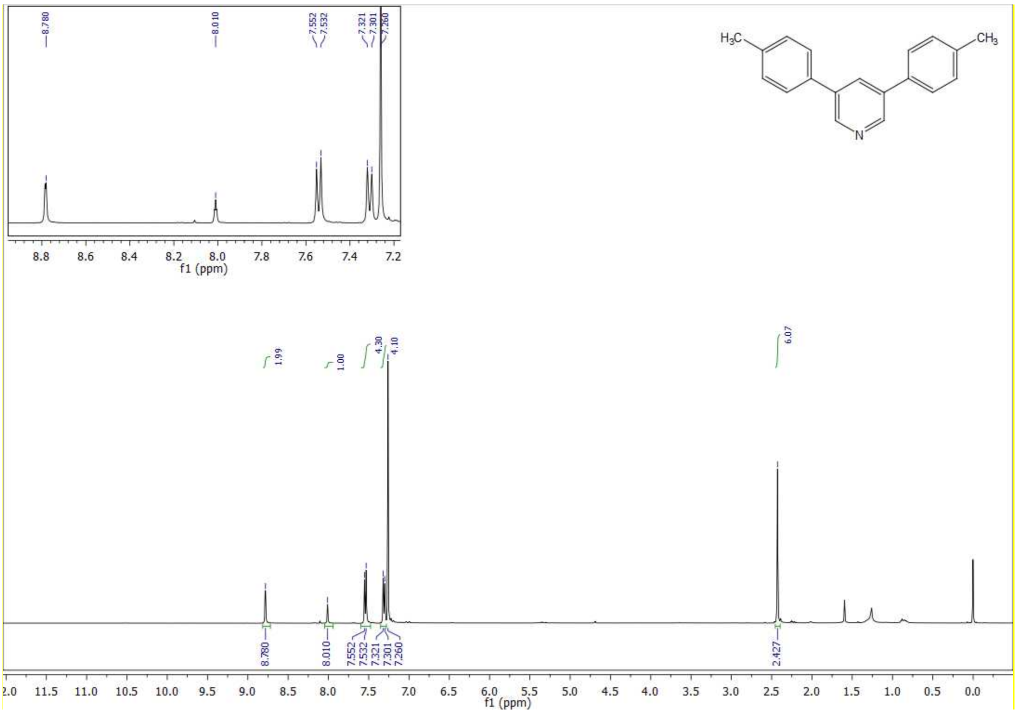

^{13}C NMR spectrum of compound **3ba** (100 MHz, $CDCl_3$)

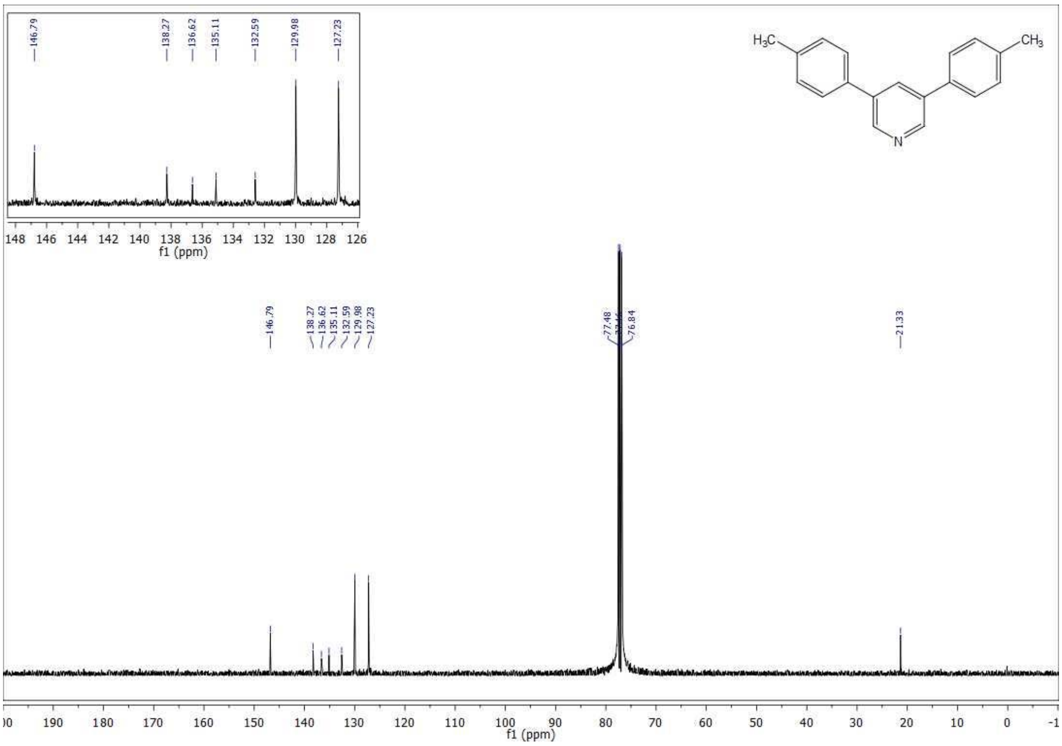

^{1}H NMR spectrum of compound **3ca** (400 MHz, $CDCl_3$)

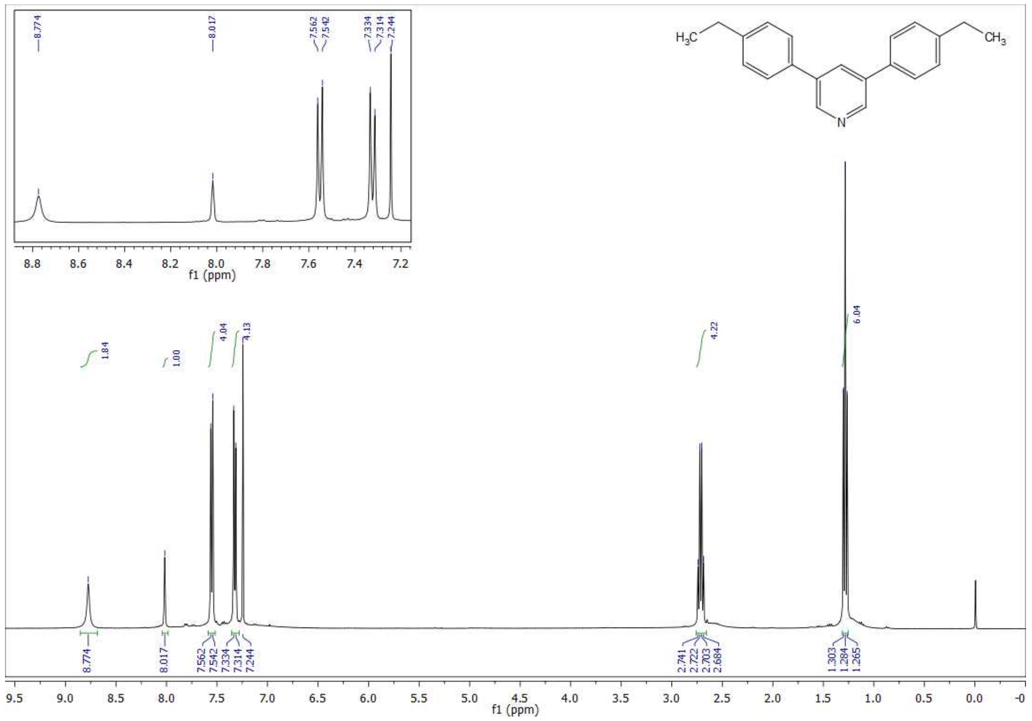

^{13}C NMR spectrum of compound **3ca** (100 MHz, $CDCl_3$)

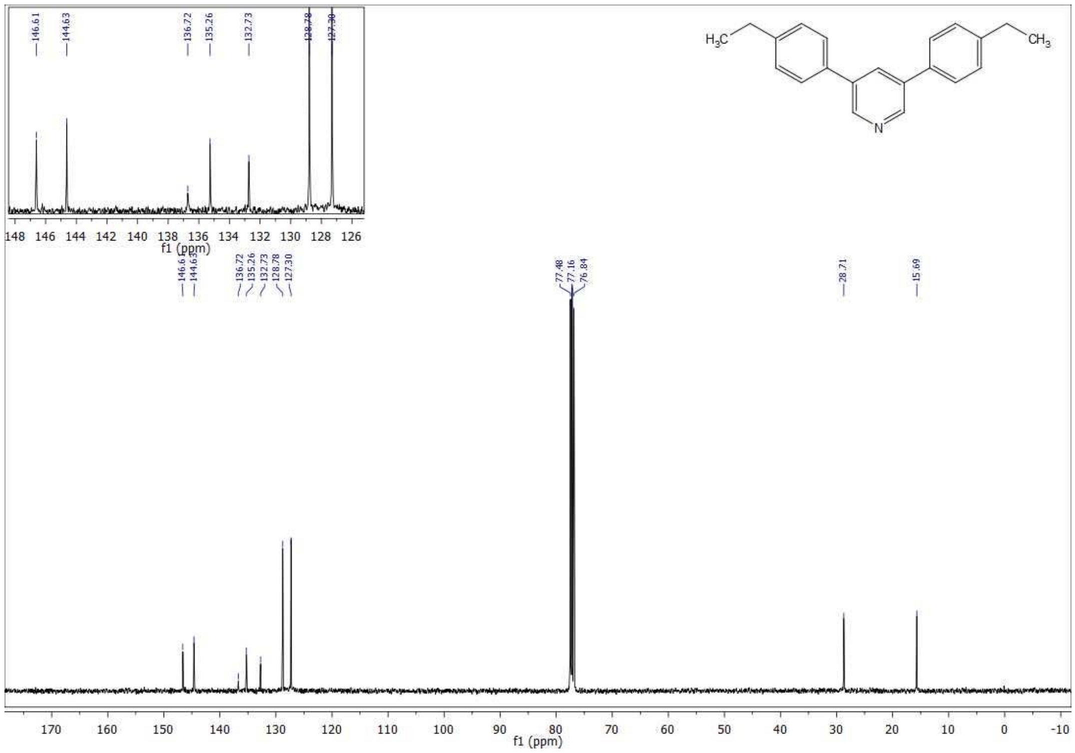

^{1}H NMR spectrum of compound **3da** (400 MHz, $CDCl_3$)

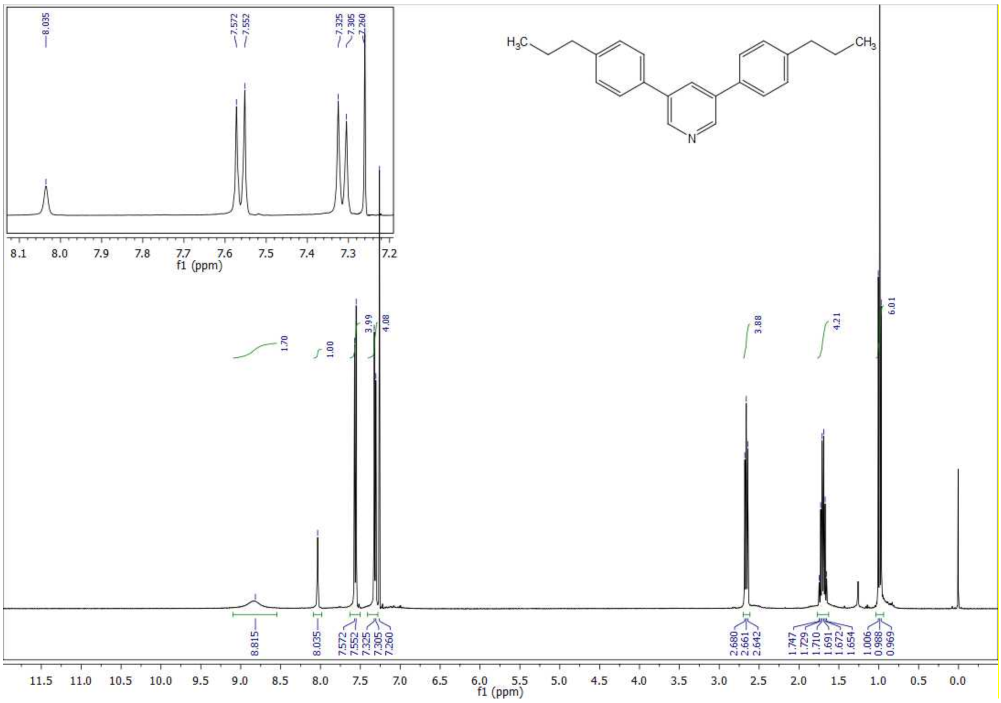

^{13}C NMR spectrum of compound **3da** (100 MHz, $CDCl_3$)

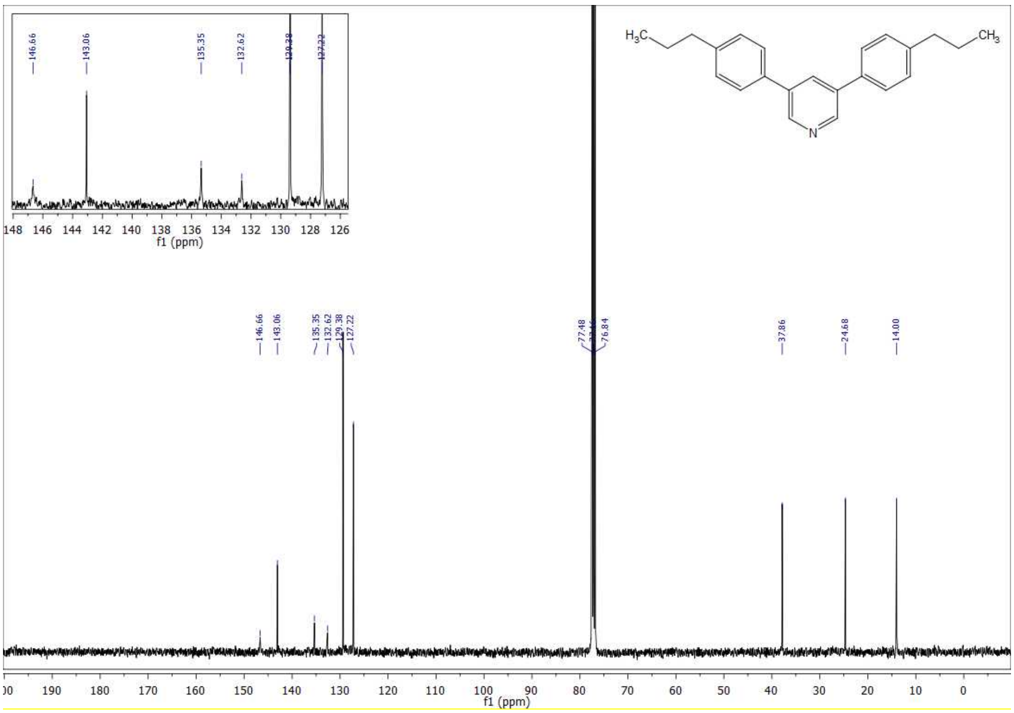

^{1}H NMR spectrum of compound **3ea** (400 MHz, $CDCl_3$)

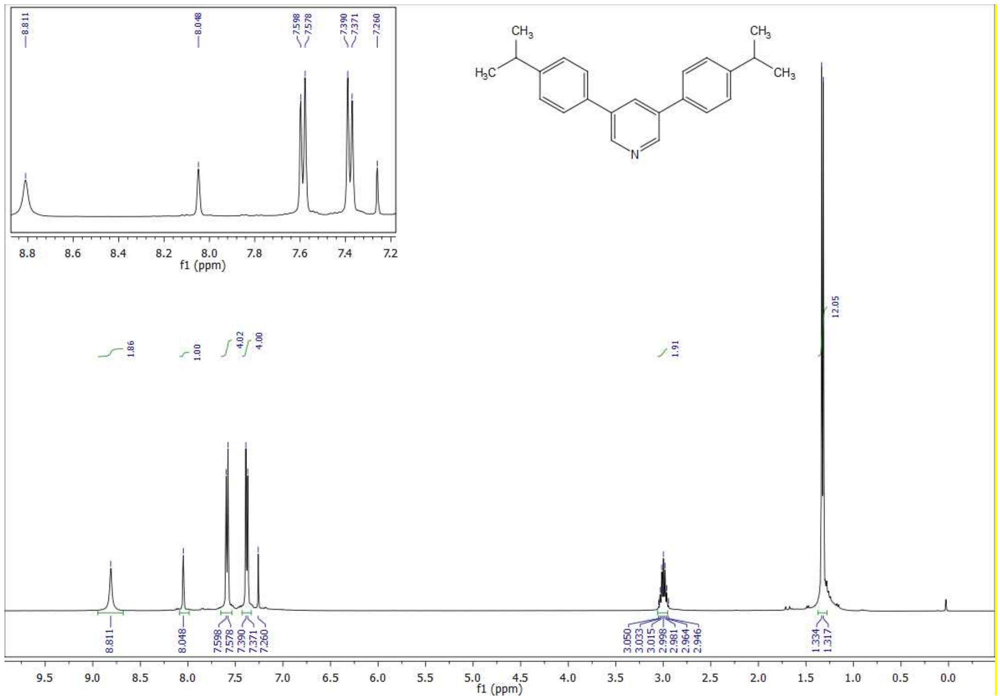

^{13}C NMR spectrum of compound **3ea** (100 MHz, $CDCl_3$)

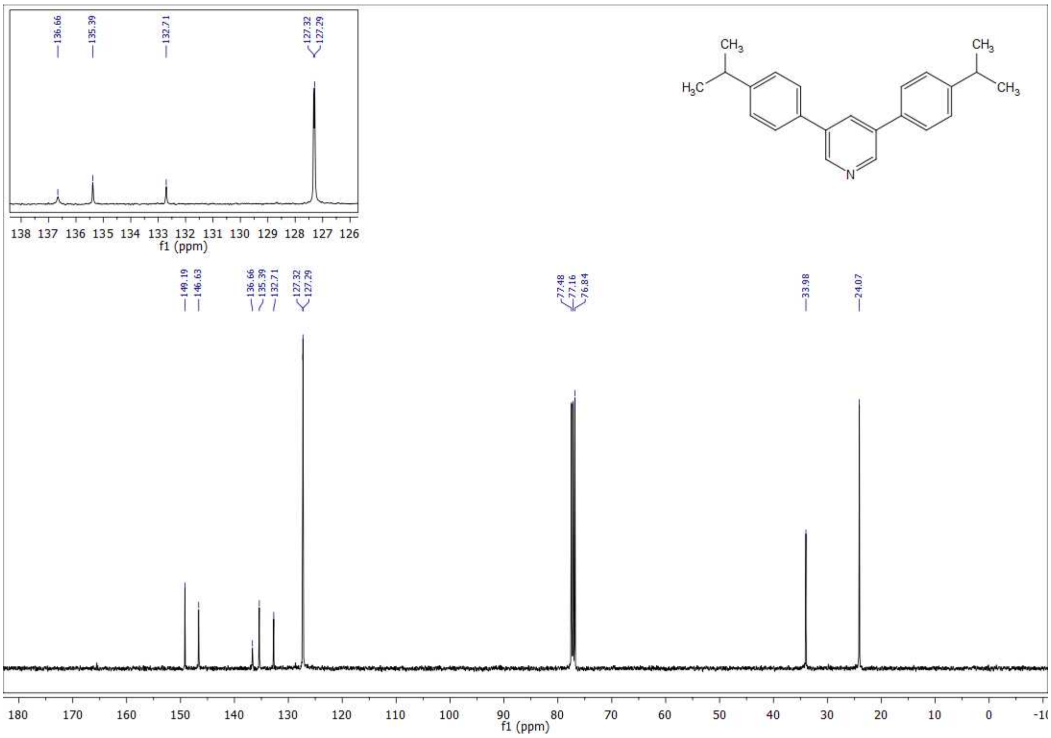

^{1}H NMR spectrum of compound **3fa** (400 MHz, $CDCl_3$)

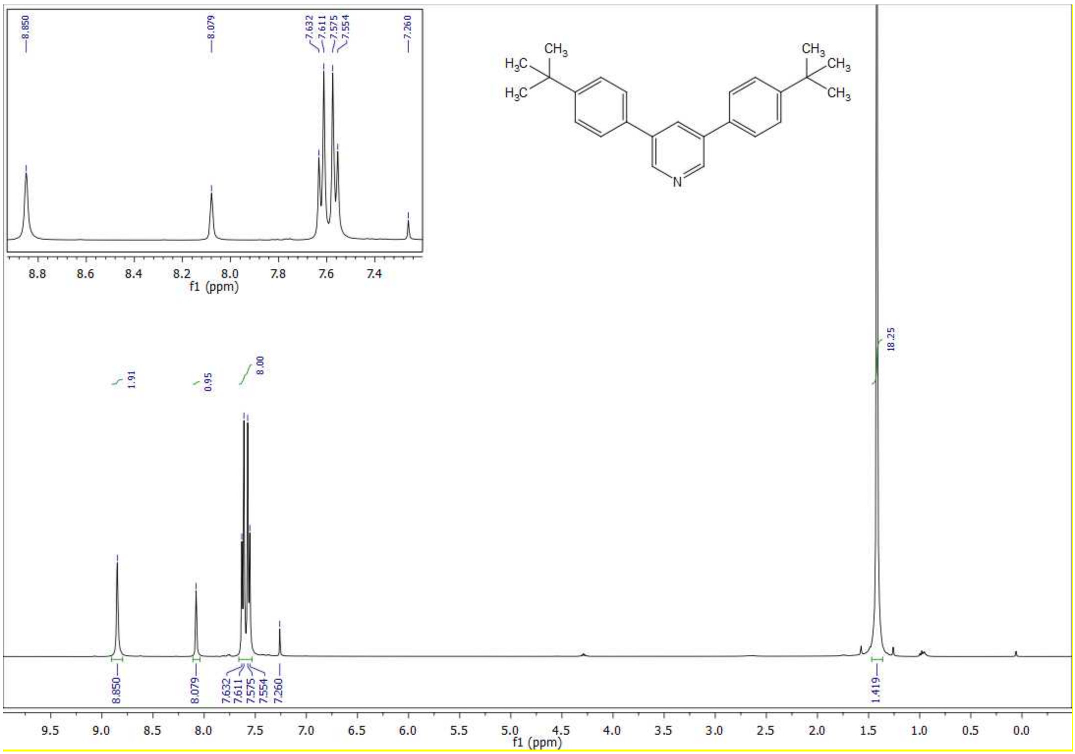

^{13}C NMR spectrum of compound **3fa** (100 MHz, $CDCl_3$)

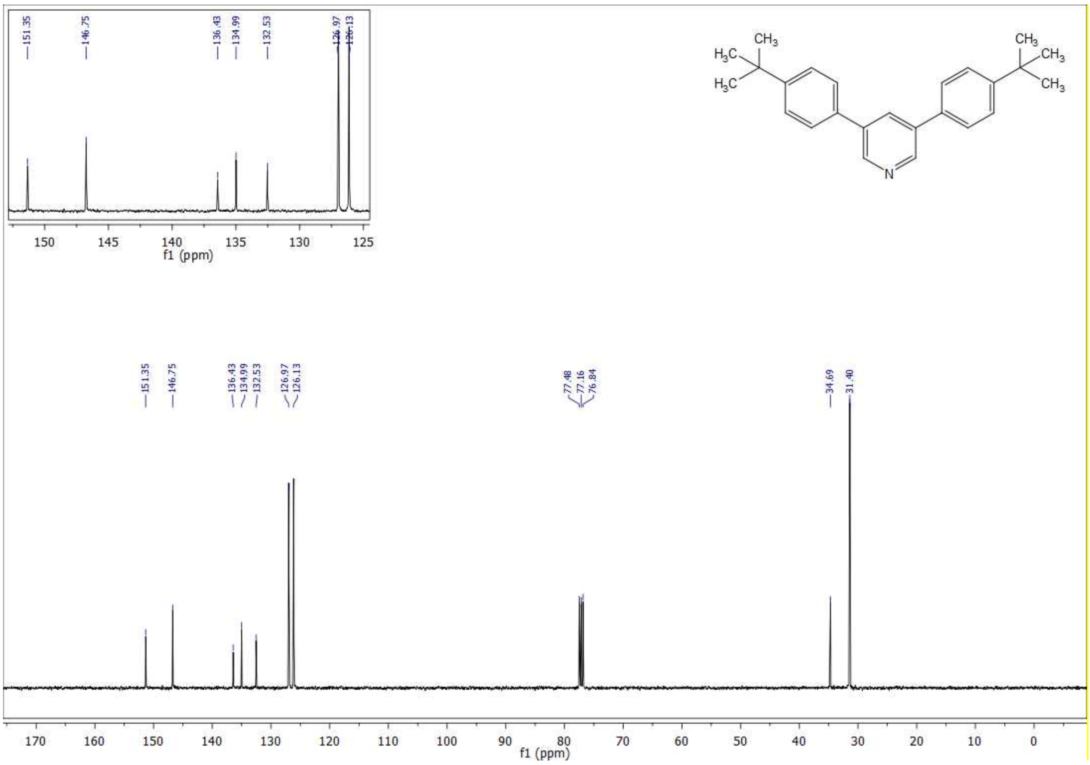

^{1}H NMR spectrum of compound **3ga** (400 MHz, $CDCl_3$)

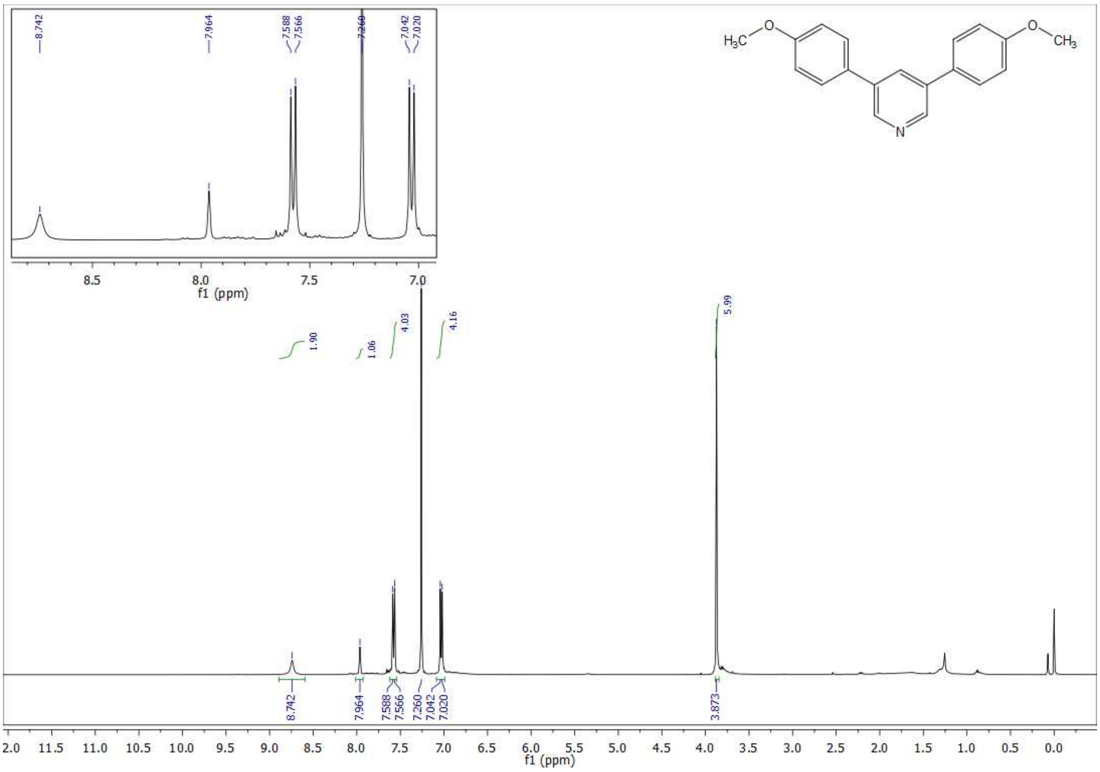

^{13}C NMR spectrum of compound **3ga** (100 MHz, $CDCl_3$)

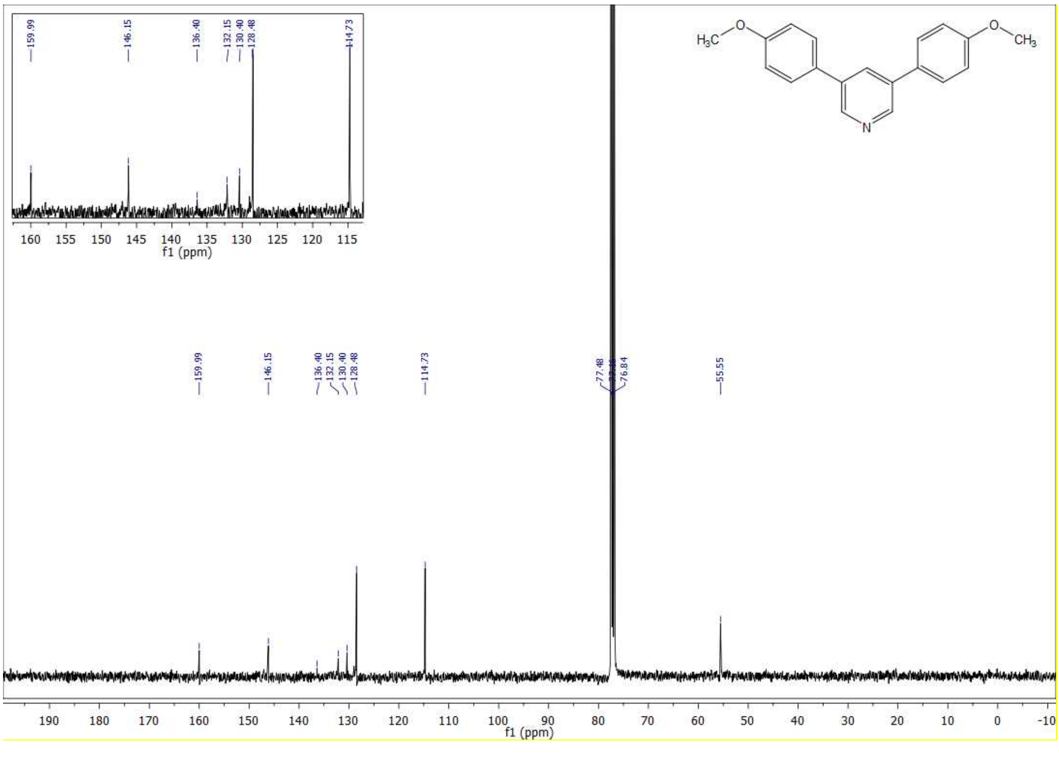

^{1}H NMR spectrum of compound **3ha** (400 MHz, $CDCl_3$)

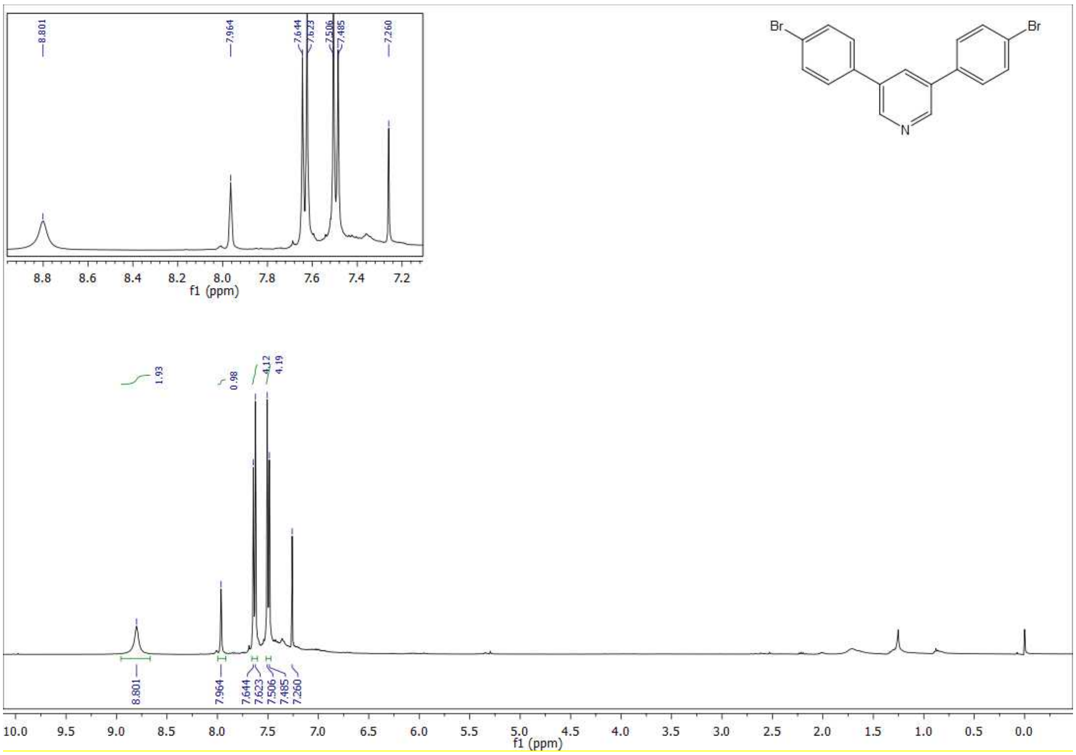

^{13}C NMR spectrum of compound **3ha** (100 MHz, $CDCl_3$)

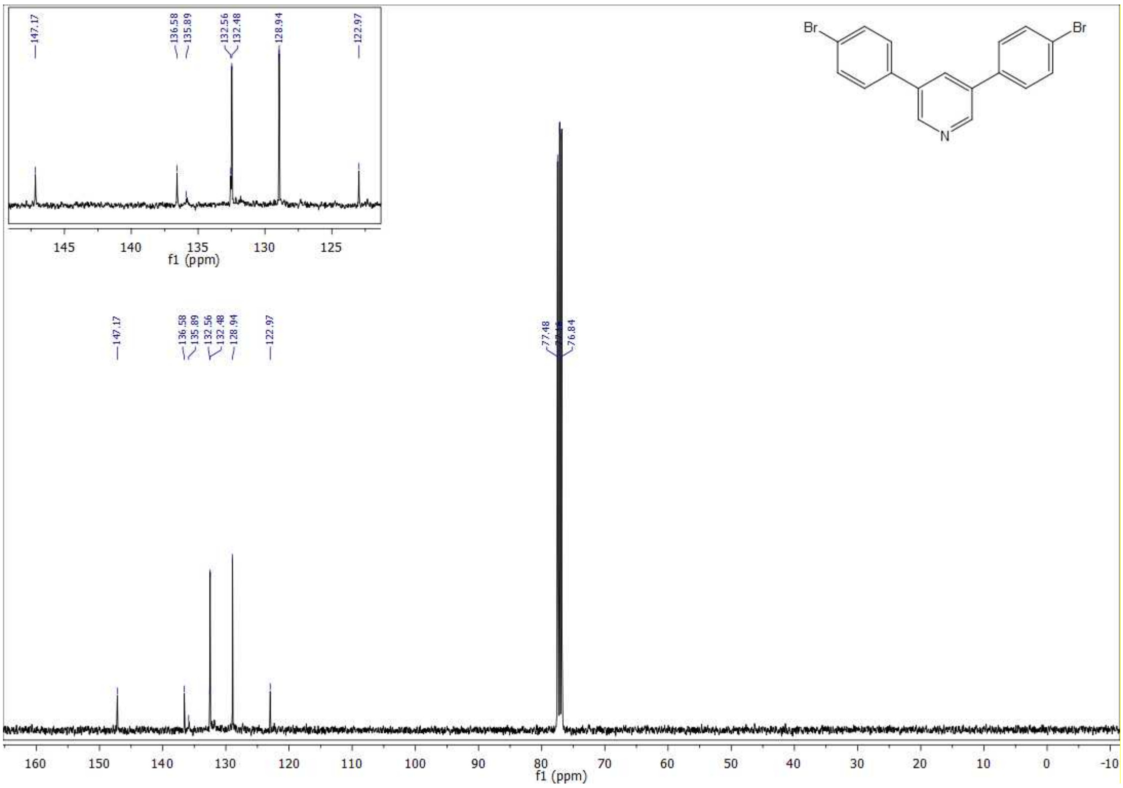

^{1}H NMR spectrum of compound **3ia** (400 MHz, $CDCl_3$)

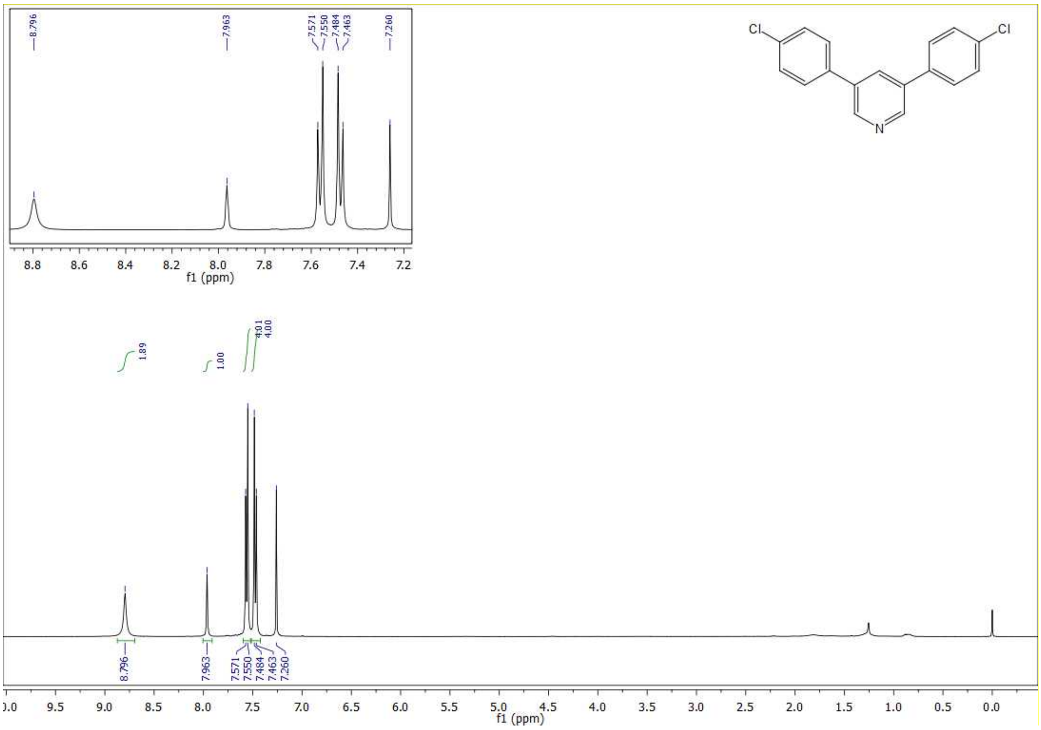

^{13}C NMR spectrum of compound **3ia** (100 MHz, $CDCl_3$)

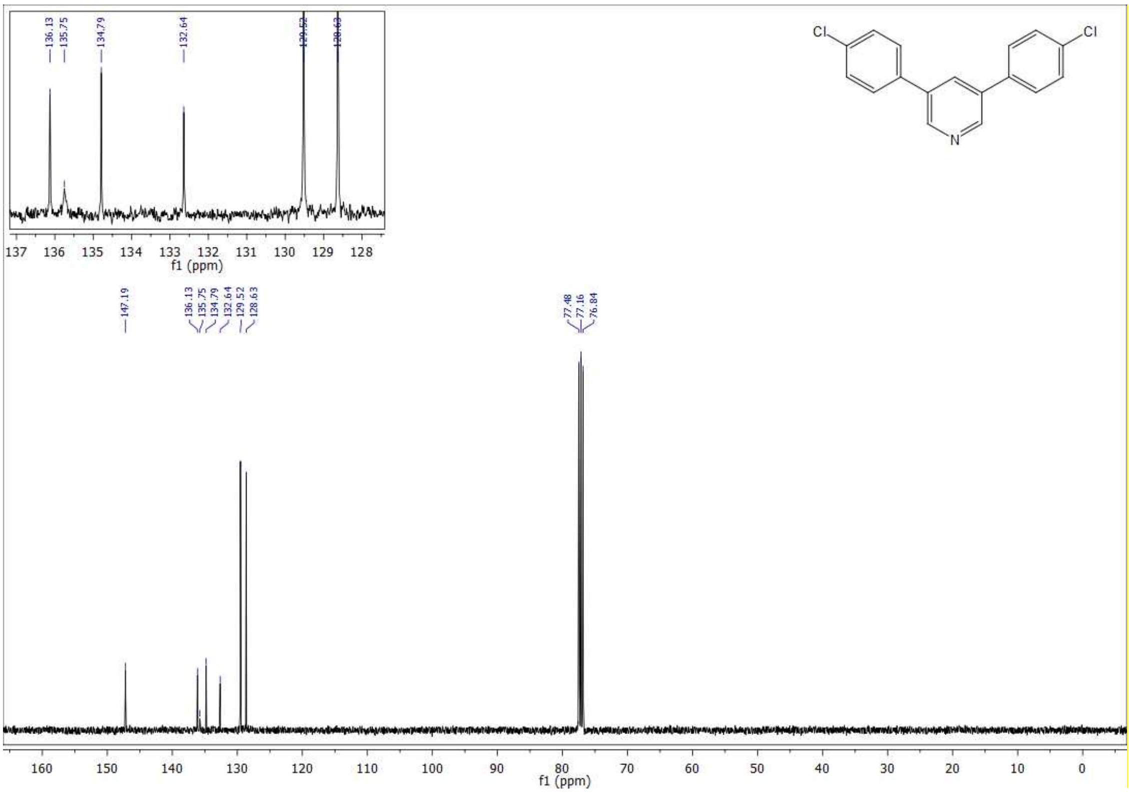

^{1}H NMR spectrum of compound **3ja** (400 MHz, $CDCl_3$)

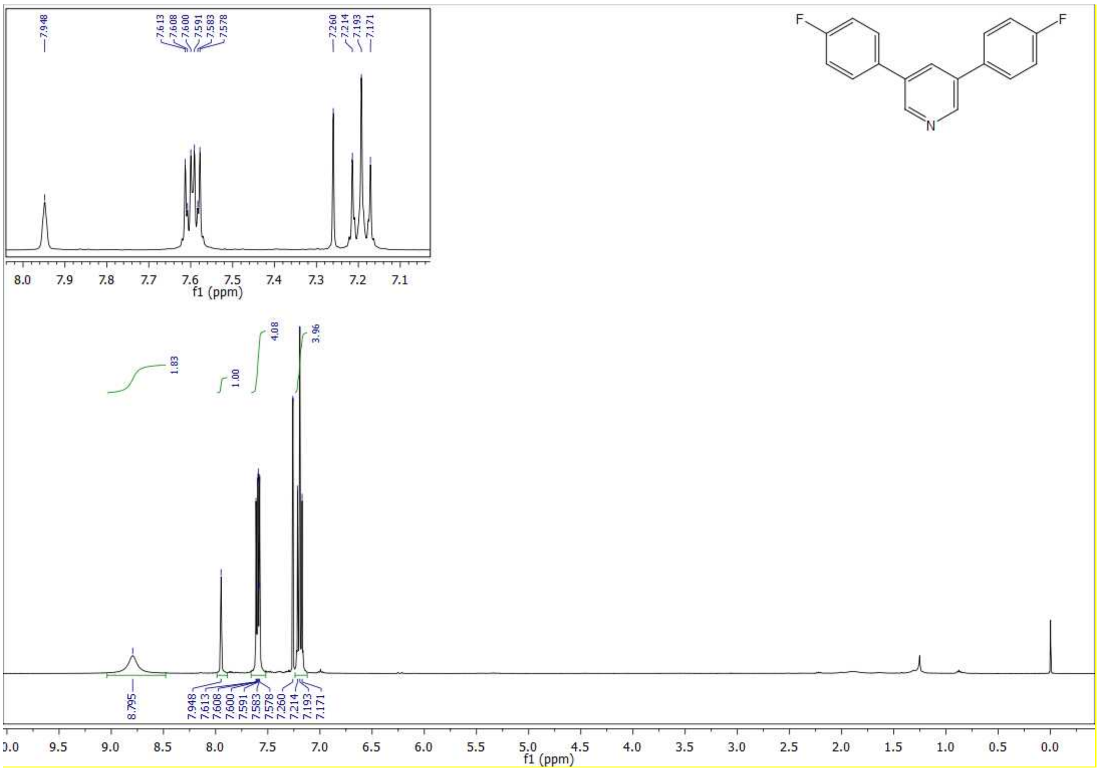

^{13}C NMR spectrum of compound **3ja** (100 MHz, $CDCl_3$)

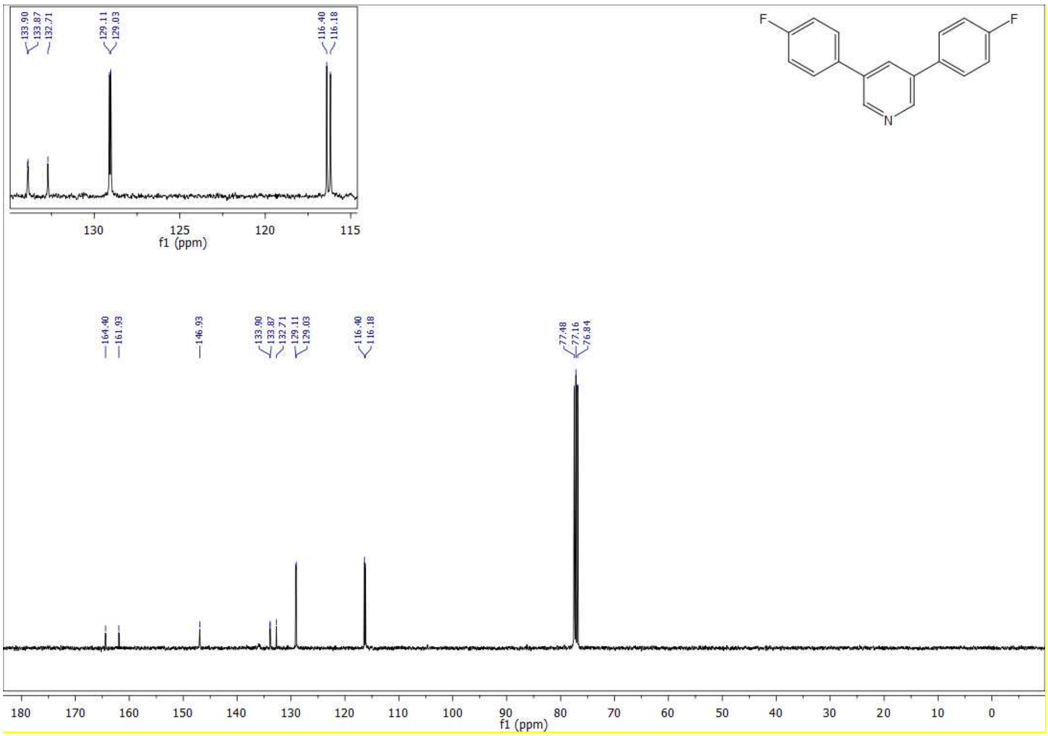

^{1}H NMR spectrum of compound **3ka** (400 MHz, $CDCl_3$)

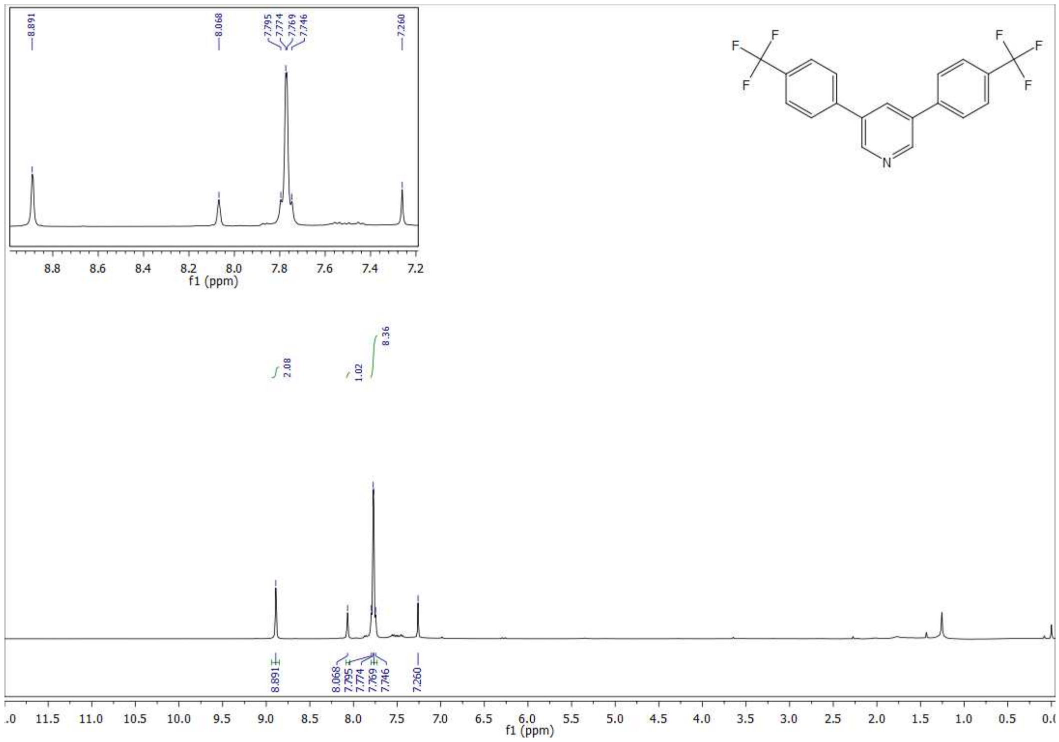

^{13}C NMR spectrum of compound **3ka** (100 MHz, $CDCl_3$)

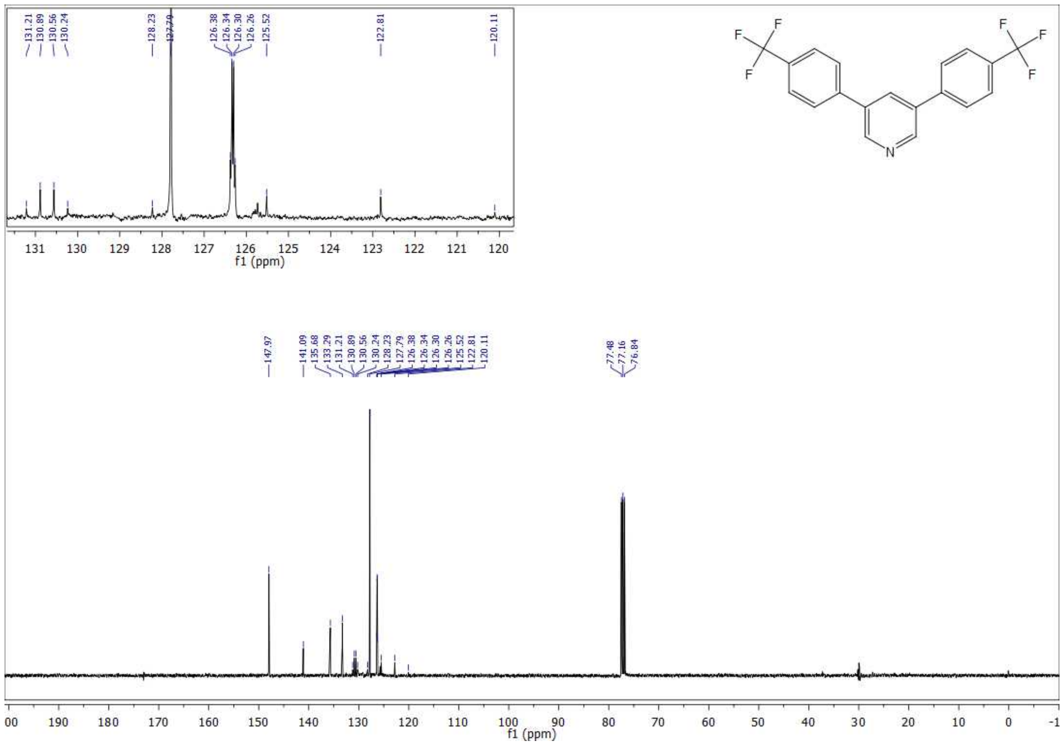

^{1}H NMR spectrum of compound **3la** (400 MHz, $CDCl_3$)

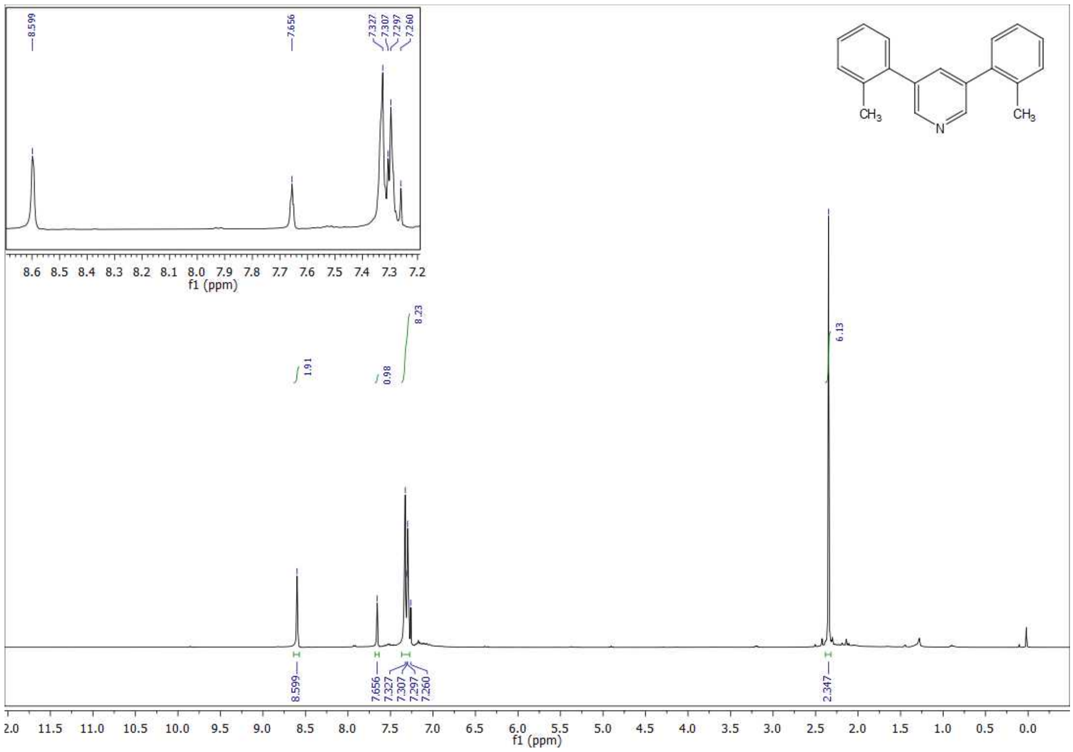

^{13}C NMR spectrum of compound **3la** (100 MHz, $CDCl_3$)

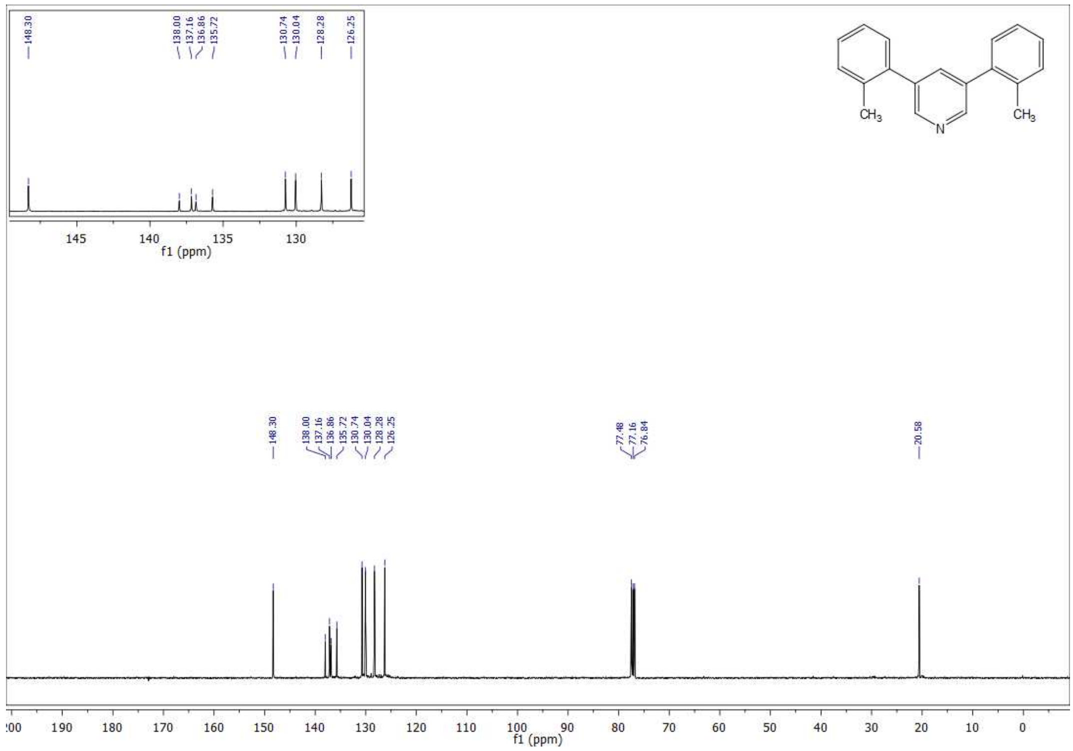

^{1}H NMR spectrum of compound **3ma** (400 MHz, $CDCl_3$)

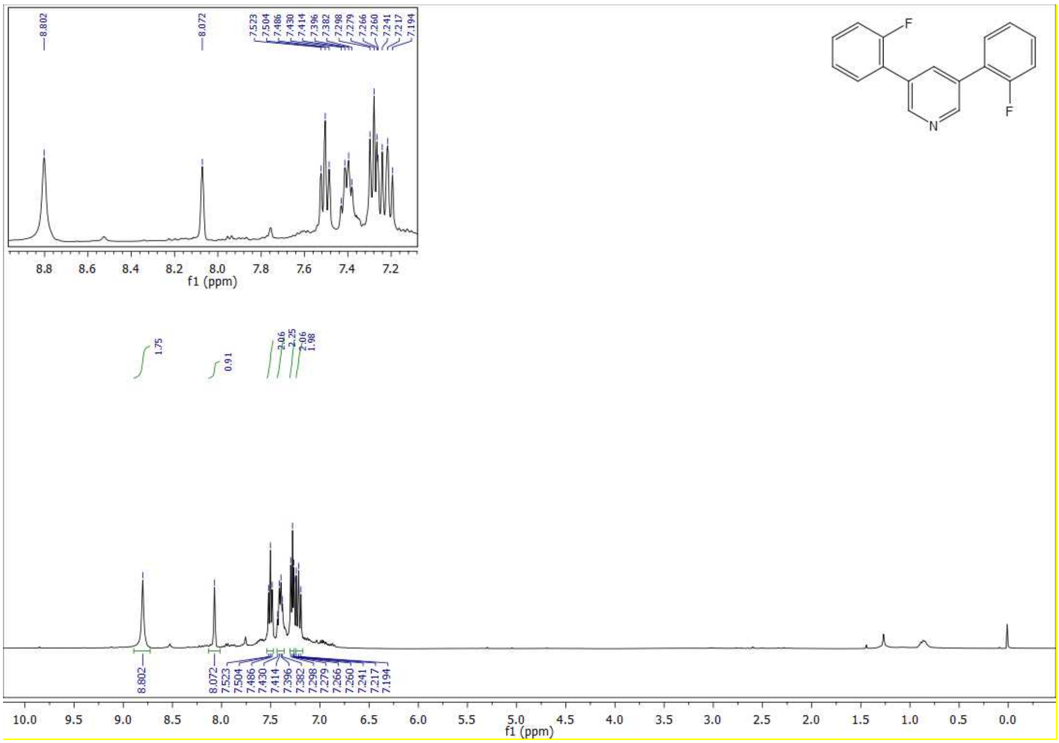

^{13}C NMR spectrum of compound **3ma** (100 MHz, $CDCl_3$)

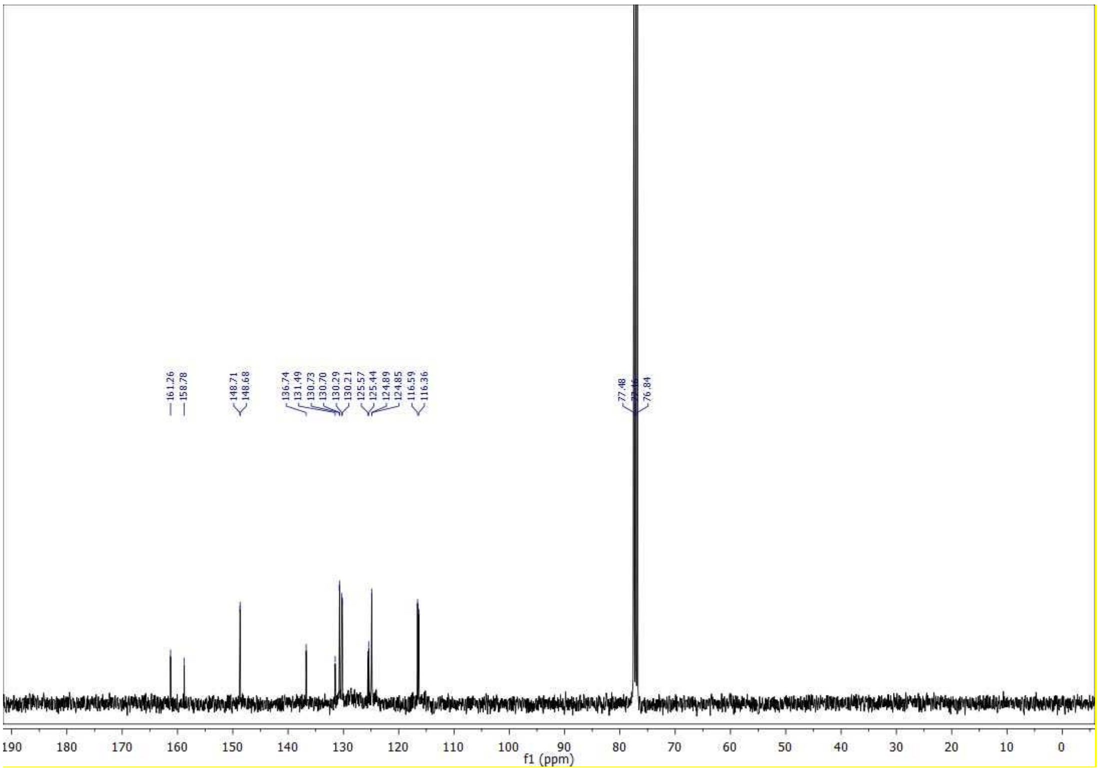

^{1}H NMR spectrum of compound **3na** (400 MHz, $CDCl_3$)

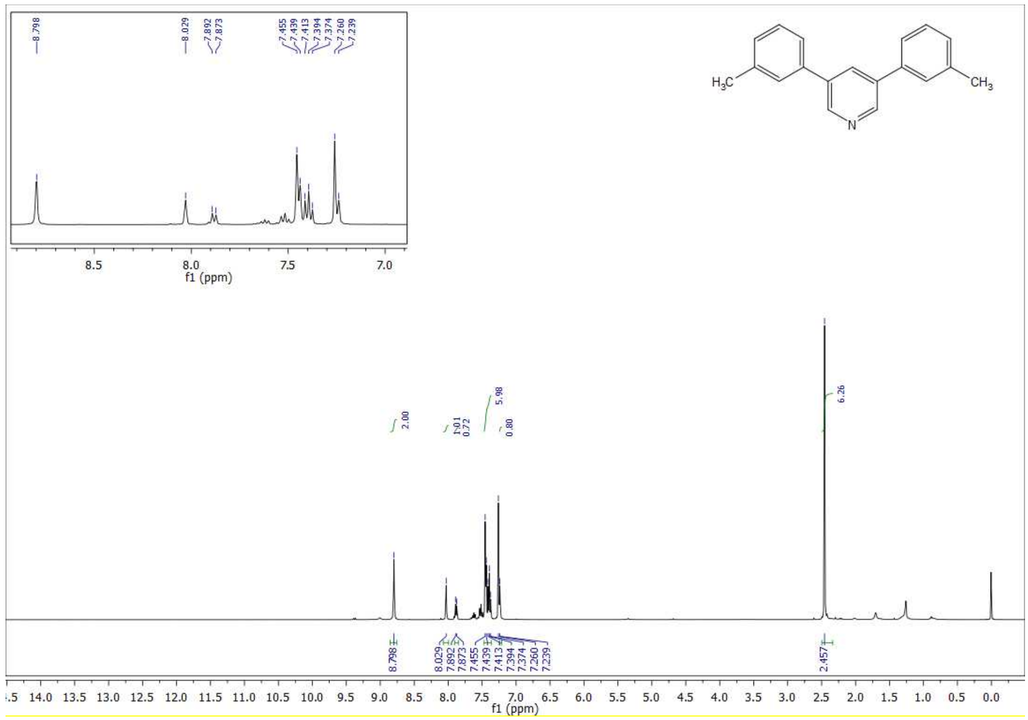

^{13}C NMR spectrum of compound **3na** (100 MHz, $CDCl_3$)

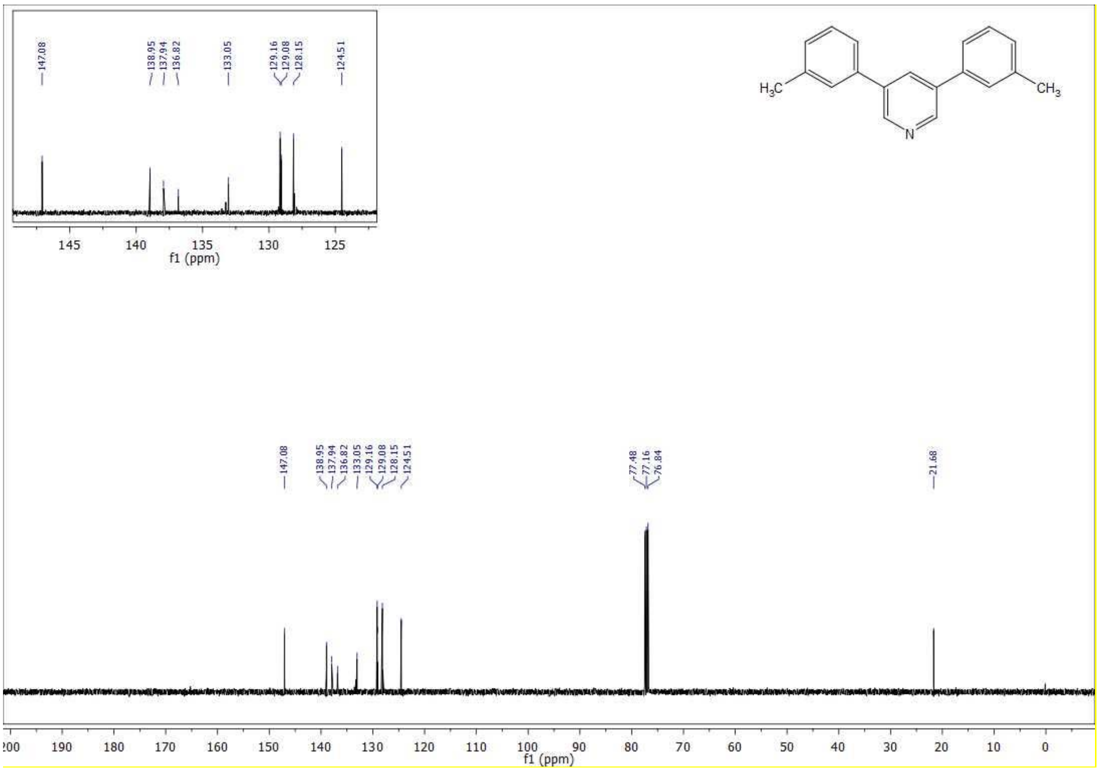

^{1}H NMR spectrum of compound **3oa** (400 MHz, $CDCl_3$)

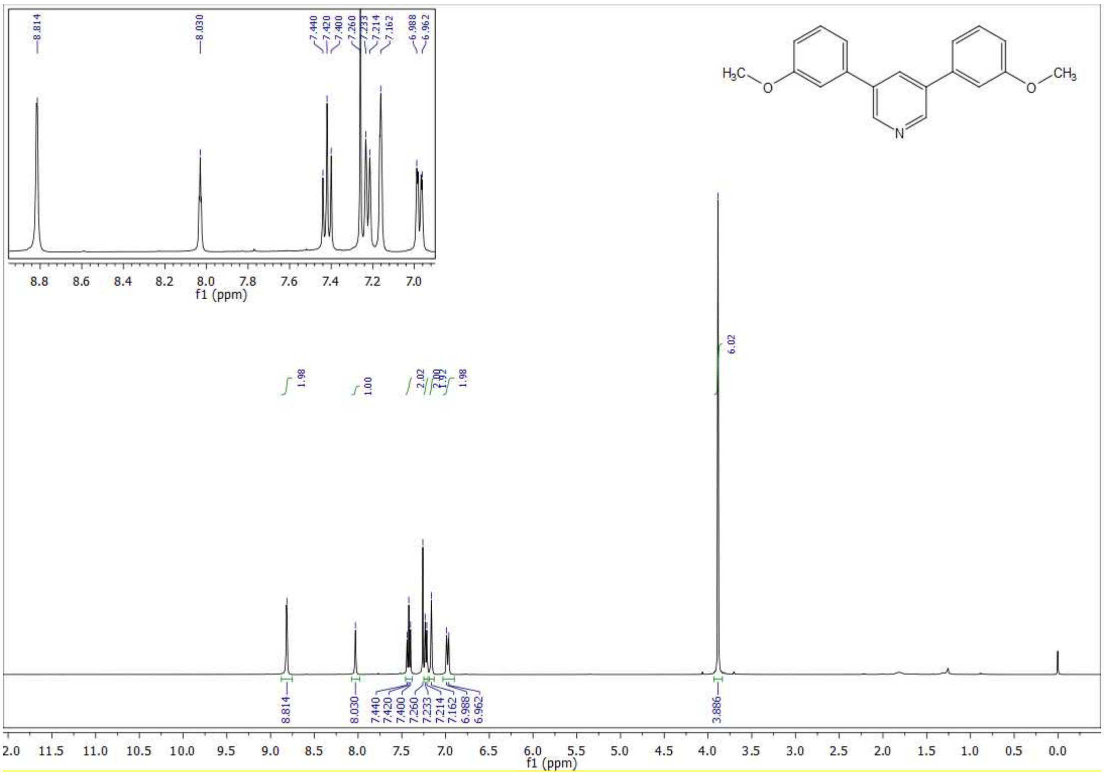

^{13}C NMR spectrum of compound **3oa** (100 MHz, $CDCl_3$)

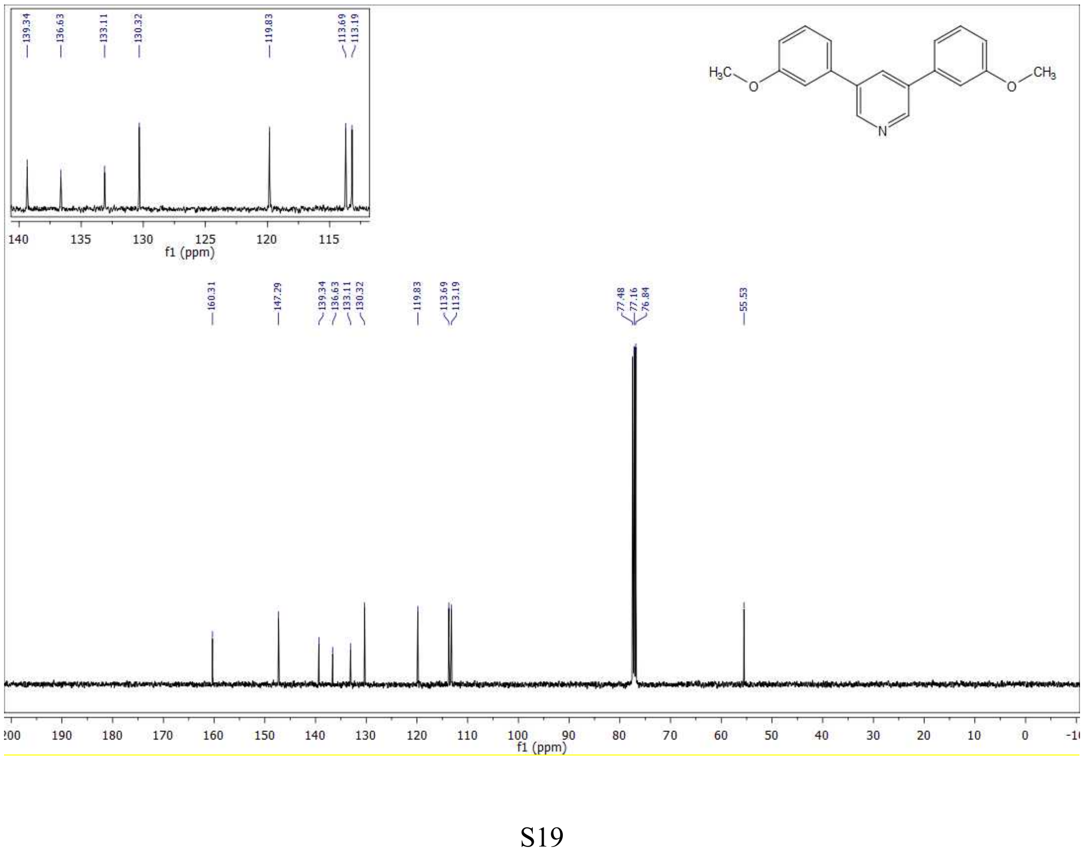

^{1}H NMR spectrum of compound **3pa** (400 MHz, $CDCl_3$)

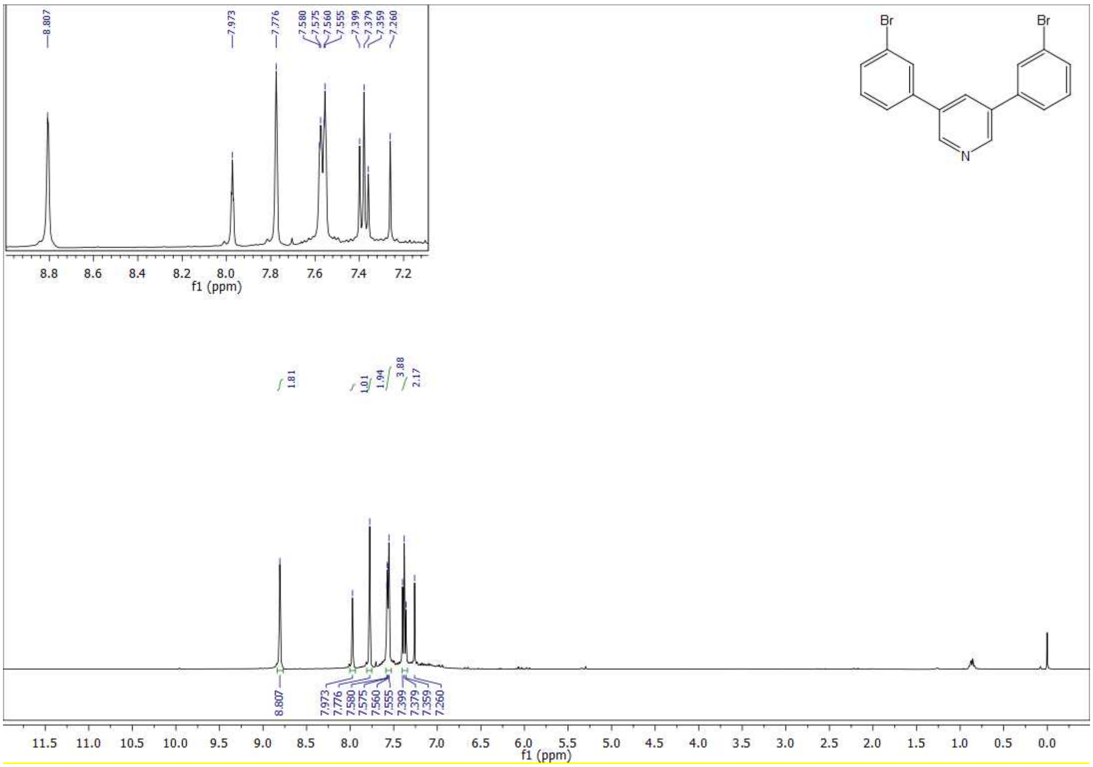

^{13}C NMR spectrum of compound **3pa** (100 MHz, $CDCl_3$)

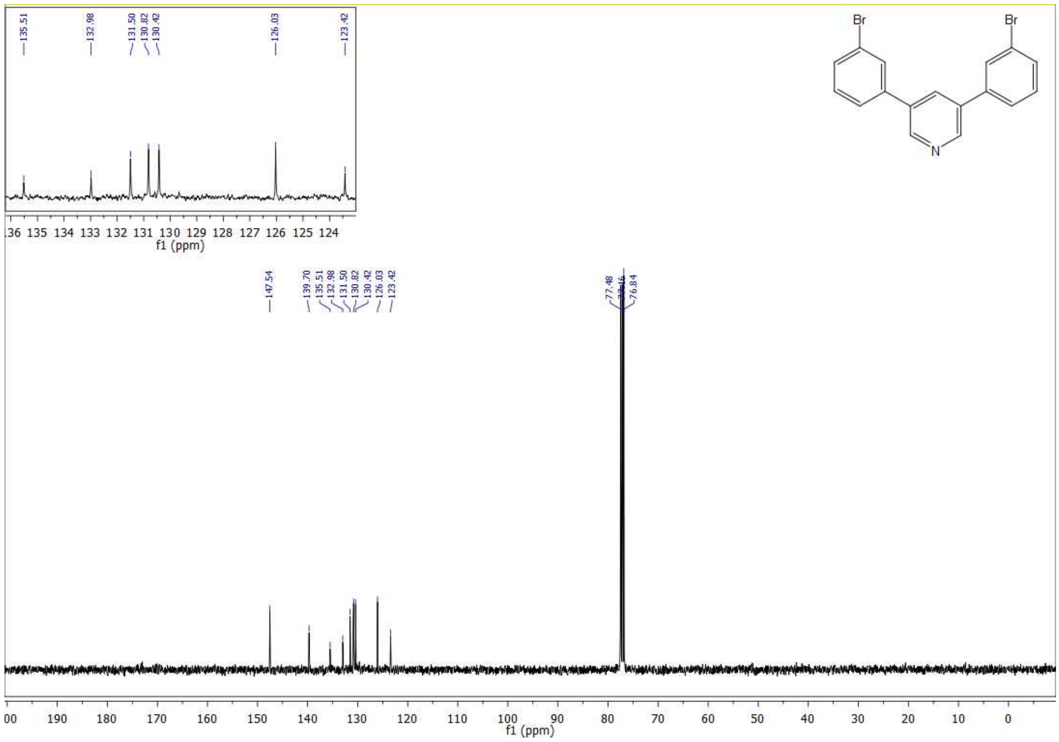

^{1}H NMR spectrum of compound **3qa** (400 MHz, $CDCl_3$)

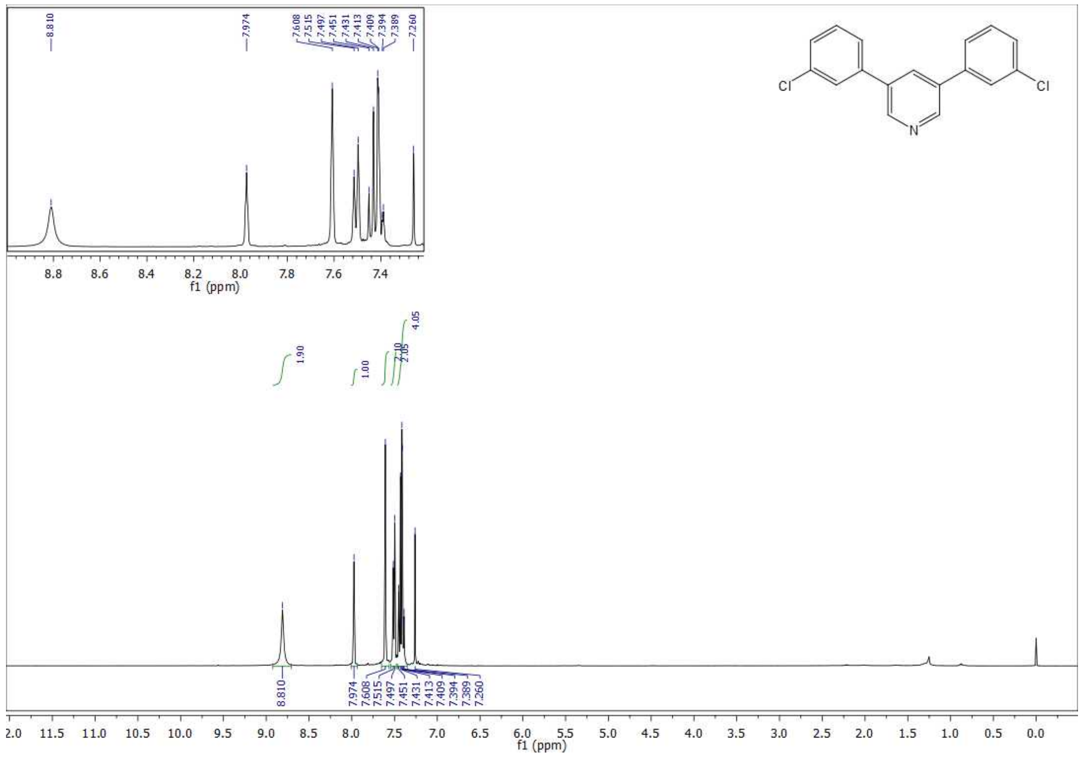

^{13}C NMR spectrum of compound **3qa** (100 MHz, $CDCl_3$)

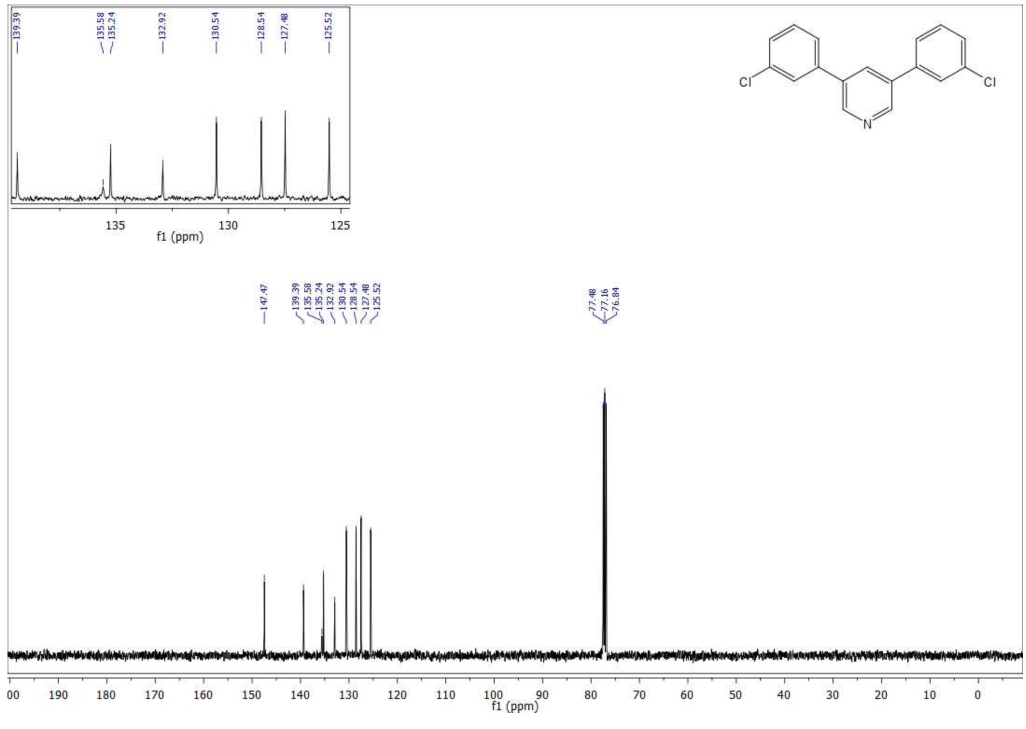

^{1}H NMR spectrum of compound **3ra** (400 MHz, $CDCl_3$)

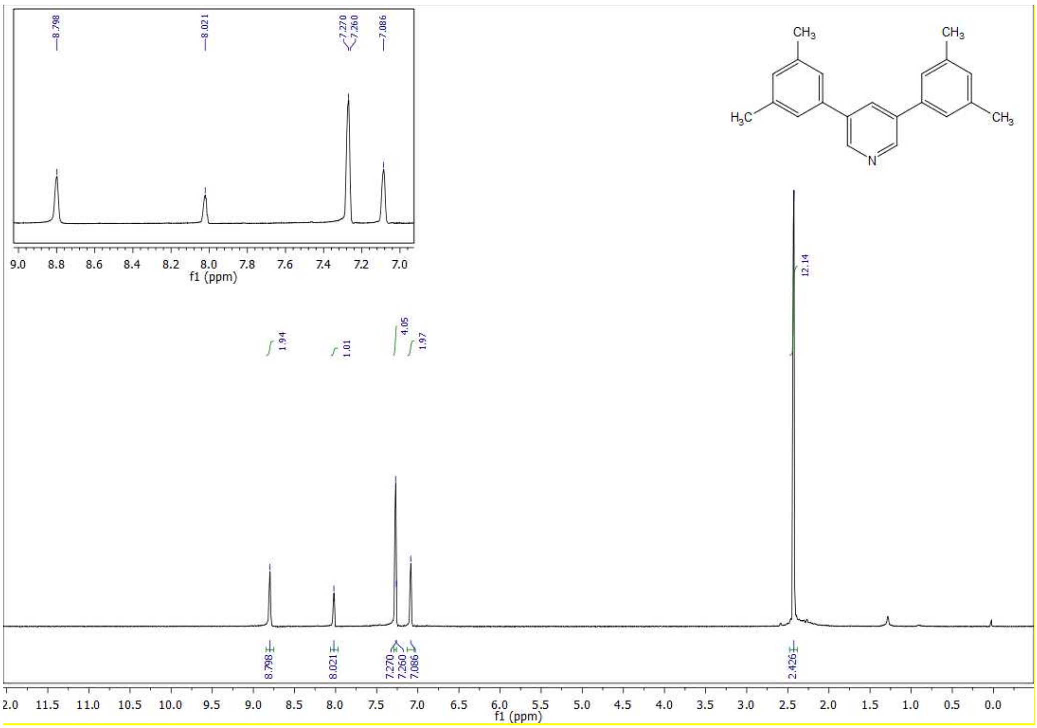

^{13}C NMR spectrum of compound **3ra** (100 MHz, $CDCl_3$)

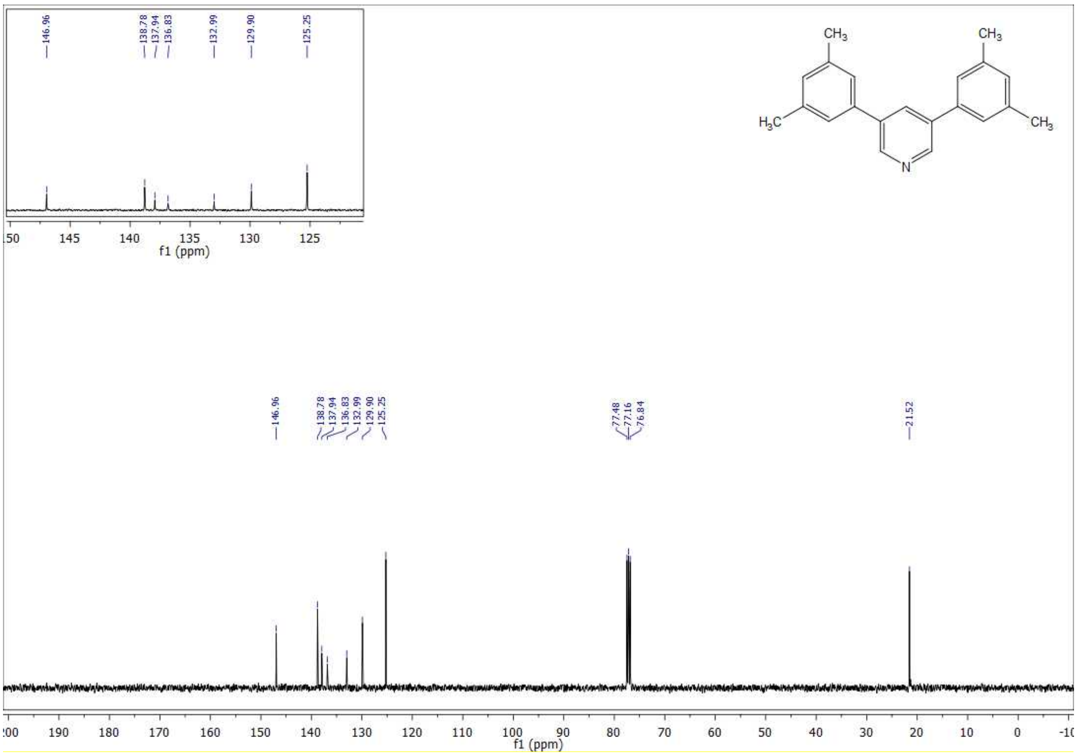

^{1}H NMR spectrum of compound **3sa** (400 MHz, $CDCl_3$)

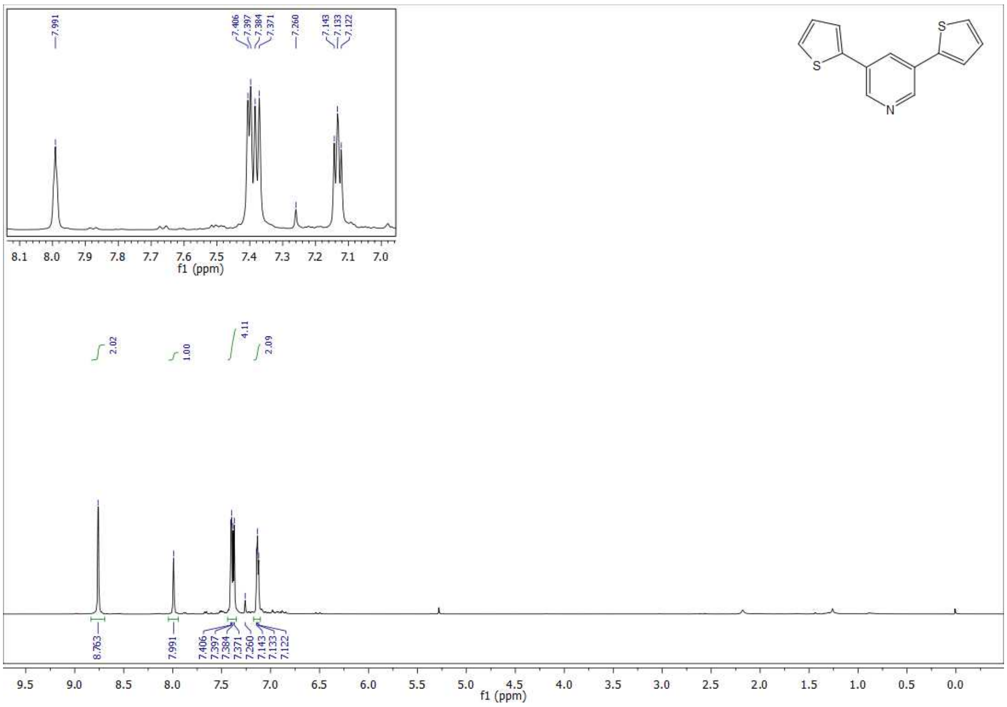

^{13}C NMR spectrum of compound **3sa** (100 MHz, $CDCl_3$)

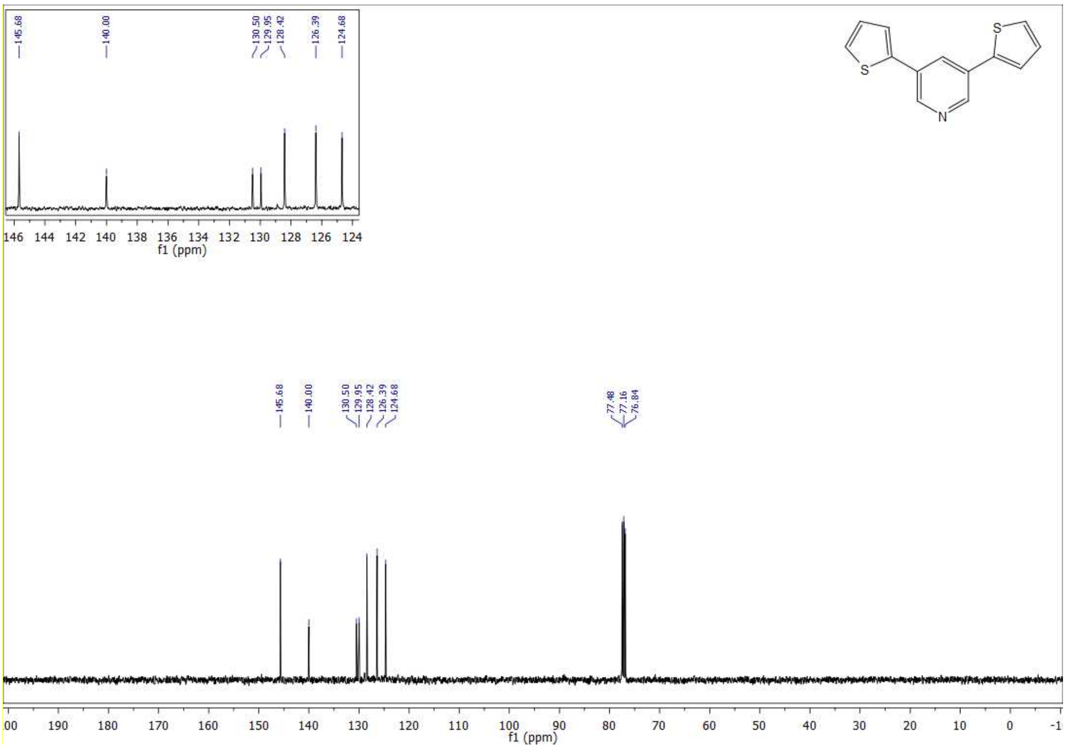

3. X-ray structural details of 3aa

Crystal Structure of $C_{17}H_{13}N$

The low temperature (173±2°K) single-crystal X-ray experiments were performed on a SuperNova diffractometer with Cu K_α radiation. Unit cell was obtained and refined by 3151 reflections with 6.4° < θ < 74.6°. No decay was observed in data collection. Raw intensities were corrected for Lorentz and polarization effects, and for absorption by empirical method. Direct phase determination yielded the positions of all non-hydrogen atoms. All non-hydrogen atoms were subjected to anisotropic refinement. All hydrogen atoms were generated geometrically with C-H bonds of 0.93 Å according to criteria described in the SHELXTL manual (Bruker, 1997). They were included in the refinement with $U_{iso}(H) = 1.2U_{eq}$ of their parent atoms. The final full-matric least-square refinement on F^2 converged with $R1 = 0.0360$ and $wR2 = 0.0941$ for 1174 observed reflections [I ≥ 2σ(I)]. The final difference electron density map shows no features. Details of crystal parameters, data collection and structure refinement are given in Table 1.

Data collection was controlled by CrysAlisPro, Agilent Technologies, Version 1.171.36.32 (Oxford, 2013). Computations were performed using the SHELXTL NT ver. 5.10 program package (Bruker, 1997) on an IBM PC 586 computer. Analytic expressions of atomic scattering factors were employed, and anomalous dispersion corrections were incorporated (*International Tables for X-ray Crystallography*, 1989). Crystal drawings were produced with XP (Bruker, 1997).

References

Bruker. (1997) SHELXTL. Structure Determination Programs, Version 5.10, Bruker AXS Inc.,6300 Enterprise Lane, Madison, WI 53719-1173, USA.

International Tables for X-ray Crystallography: (1989) Vol. C (Kluwer Academic Publishers, Dordrecht) Tables 4.2.6.8 and 6.1.1.4.

Oxford. (2013) CrysAlisPro, Agilent Technologies, Version 1.171.36.32, Oxford Diffraction Ltd., 68 Milton Park, Abingdon, Oxfordshire, OX14 4RX, UK.

Table 1. Details of Data Collection, Processing and Structure Refinement

Sample code	**2019-11-8**
Molecular formula	$C_{17}H_{13}N$
Molecular weight	231.28
Color and habit	colorless block
Crystal size	0.1 × 0.2 × 0.2 mm
Crystal system	monoclinic
Space group	*C*2/*c* (No. 15)
Unit cell parameters	a = 26.8340(5) Å α = 90.00°
	b = 6.48600(10) Å β = 102.282(2)°
	c = 7.25250(10) Å γ = 90.00°
	V = 1233.37(4) $Å^3$ Z = 4 F(000) = 488
Density (calcd)	1.246 g/cm^3
Diffractometer	SuperNova, Dual, Cu at home/near, AtlasS2
Radiation	Cu K_{α}, λ = 1.54178 Å
Temperature	173±2K
Scan type	ω-scan
Data collection range	$-32 < h < 33$, $-7 < k < 8$, $-9 < l < 6$; $\theta_{max} = 74.8°$
Reflections measured	Total: 4309 Unique (n): 1242 Observed [I ≥ 2σ(I)]: 1174
Absorption coefficient	0.555 mm^{-1}
Minimum and maximum transmission	0.793, 1.000
No. of variables, p	83
Weighting scheme	$w = \dfrac{1}{\sigma^2(F_o^2) + (0.0508P)^2 + 0.5344P}$ $P = (F_o^2 + 2F_c^2)/3$
$R1 = \dfrac{\Sigma\|\|F_o\|-\|F_c\|\|}{\Sigma\|F_o\|}$ (for all reflections)	0.0374 0.0360 (for observed data)
$wR2 = \sqrt{\dfrac{\Sigma[w(F_o^2 - F_c^2)^2]}{\Sigma w(F_o^2)^2}}$ (for all reflections)	0.0957 0.0941 (for observed data)
Goof = S = $\sqrt{\dfrac{\Sigma[w(F_o^2 - F_c^2)^2]}{n - p}}$	1.062
Largest and mean Δ/σ	0.000, 0.000
Residual extrema in final difference map	-0.188 to 0.168 e $Å^{-3}$

Table 2. Atomic coordinates and equivalent isotropic temperature factors* (Å^2)

Atoms	*x*	*y*	*z*	$U_{eq.}$
N(1)	0.0000	-0.26567(17)	0.7500	0.0275(3)
C(1)	0.04235(4)	-0.15766(15)	0.74950(14)	0.0251(2)
C(2)	0.04510(3)	0.05737(14)	0.75348(12)	0.0219(2)
C(3)	0.0000	0.16560(19)	0.7500	0.0216(3)
C(4)	0.09459(3)	0.16136(14)	0.75782(13)	0.0223(2)
C(5)	0.14038(4)	0.06931(16)	0.84979(14)	0.0264(2)
C(6)	0.18682(4)	0.16265(17)	0.84778(15)	0.0318(3)
C(7)	0.18844(4)	0.34874(18)	0.75494(16)	0.0339(3)
C(8)	0.14333(4)	0.44288(16)	0.66556(14)	0.0305(3)
C(9)	0.09681(4)	0.35042(14)	0.66692(13)	0.0250(2)

*$U_{eq.}$ defined as one third of the trace of the orthogonalized **U** tensor.

Table 3. Bond lengths (Å) and bond angles (º)

N(1)-C(1)#1	1.3357(11)	C(4)-C(9)	1.3994(14)
N(1)-C(1)	1.3357(11)	C(4)-C(5)	1.4008(13)
C(1)-C(2)	1.3967(14)	C(5)-C(6)	1.3882(14)
C(2)-C(3)	1.3946(11)	C(6)-C(7)	1.3873(16)
C(2)-C(4)	1.4839(12)	C(7)-C(8)	1.3871(15)
C(3)-C(2)#1	1.3946(11)	C(8)-C(9)	1.3869(13)
C(1)#1-N(1)-C(1)	116.73(11)	C(9)-C(4)-C(2)	121.05(8)
N(1)-C(1)-C(2)	124.40(9)	C(5)-C(4)-C(2)	120.45(9)
C(3)-C(2)-C(1)	117.42(9)	C(6)-C(5)-C(4)	120.50(10)
C(3)-C(2)-C(4)	122.74(9)	C(7)-C(6)-C(5)	120.36(9)
C(1)-C(2)-C(4)	119.83(8)	C(8)-C(7)-C(6)	119.70(9)
C(2)#1-C(3)-C(2)	119.56(12)	C(9)-C(8)-C(7)	120.24(10)
C(9)-C(4)-C(5)	118.48(9)	C(8)-C(9)-C(4)	120.72(9)

Symmetry transformation code: #1 (-*x*, *y*, 1.5-*z*).

Table 4. Anisotropic thermal parameters* (Å^2)

Atoms	U_{11}	U_{22}	U_{33}	U_{23}	U_{13}	U_{12}
N(1)	0.0293(6)	0.0199(6)	0.0319(6)	0.000	0.0035(5)	0.000
C(1)	0.0247(5)	0.0226(5)	0.0273(5)	-0.0012(3)	0.0036(4)	0.0031(3)
C(2)	0.0231(5)	0.0227(5)	0.0192(4)	-0.0001(3)	0.0030(3)	0.0006(3)
C(3)	0.0238(6)	0.0186(6)	0.0221(6)	0.000	0.0039(5)	0.000
C(4)	0.0219(5)	0.0239(5)	0.0216(4)	-0.0038(3)	0.0055(3)	0.0009(3)
C(5)	0.0257(5)	0.0279(5)	0.0256(5)	-0.0004(4)	0.0056(4)	0.0024(4)
C(6)	0.0216(5)	0.0416(6)	0.0321(5)	-0.0026(4)	0.0051(4)	0.0031(4)
C(7)	0.0249(5)	0.0435(6)	0.0351(6)	-0.0038(4)	0.0106(4)	-0.0081(4)
C(8)	0.0331(5)	0.0302(5)	0.0295(5)	-0.0003(4)	0.0099(4)	-0.0060(4)
C(9)	0.0256(5)	0.0246(5)	0.0246(5)	-0.0023(3)	0.0048(4)	0.0004(3)

*The exponent takes the form: $-2\pi^2\Sigma\Sigma U_{ij}h_ih_j\mathbf{a}_i^*\mathbf{a}_j^*$

Table 5. Coordinates and isotropic temperature factors* (Å^2) for H atoms

Atoms	*x*	*y*	*z*	$U_{eq.}$
H(1)	0.0720	-0.2305	0.7463	0.030
H(3)	0.0000	0.3090	0.7500	0.026
H(5)	0.1397	-0.0553	0.9127	0.032
H(6)	0.2170	0.1001	0.9090	0.038
H(7)	0.2196	0.4101	0.7526	0.041
H(8)	0.1443	0.5685	0.6045	0.037
H(9)	0.0668	0.4147	0.6068	0.030

*The exponent takes the form: $-8\pi^2 U \sin^2\theta/\lambda^2$

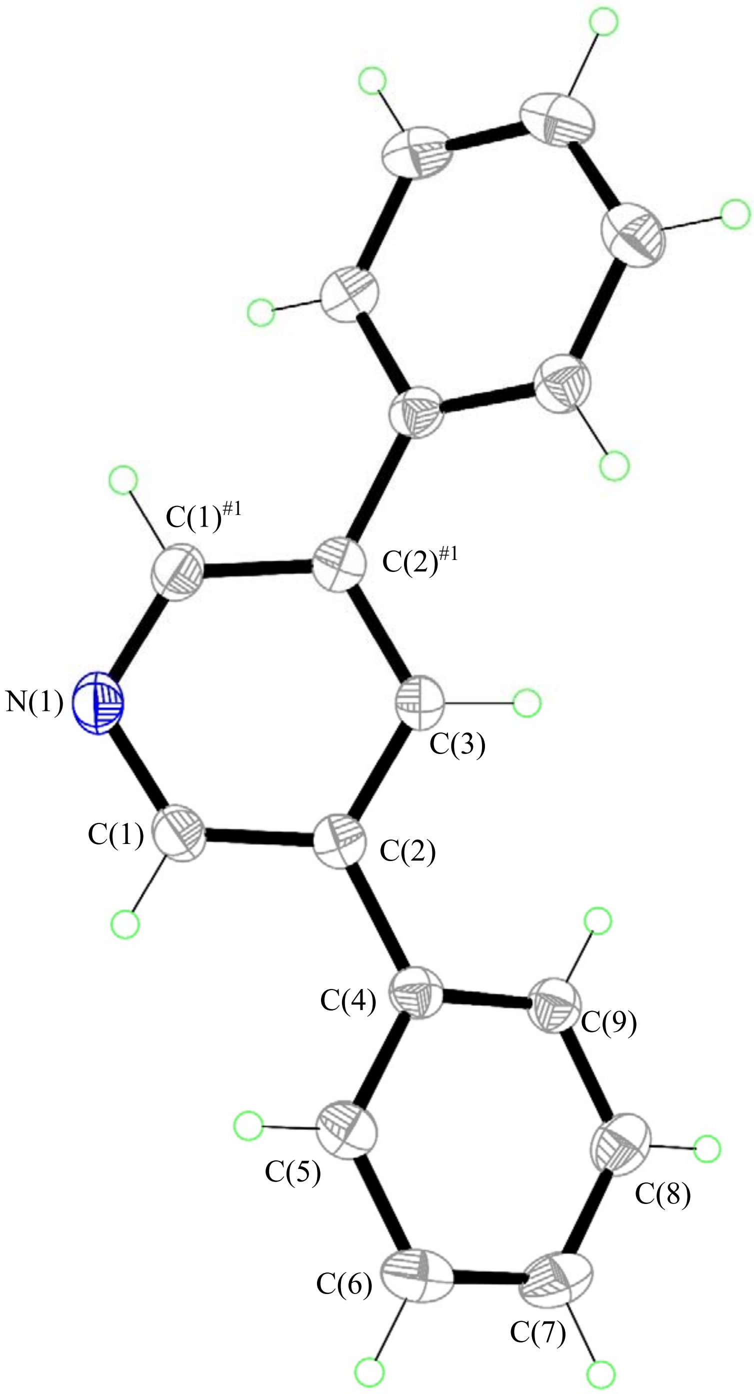

ORTEP drawing of $C_{17}H_{13}N$ with 50% probability ellipsoids, showing the atomic numbering scheme.

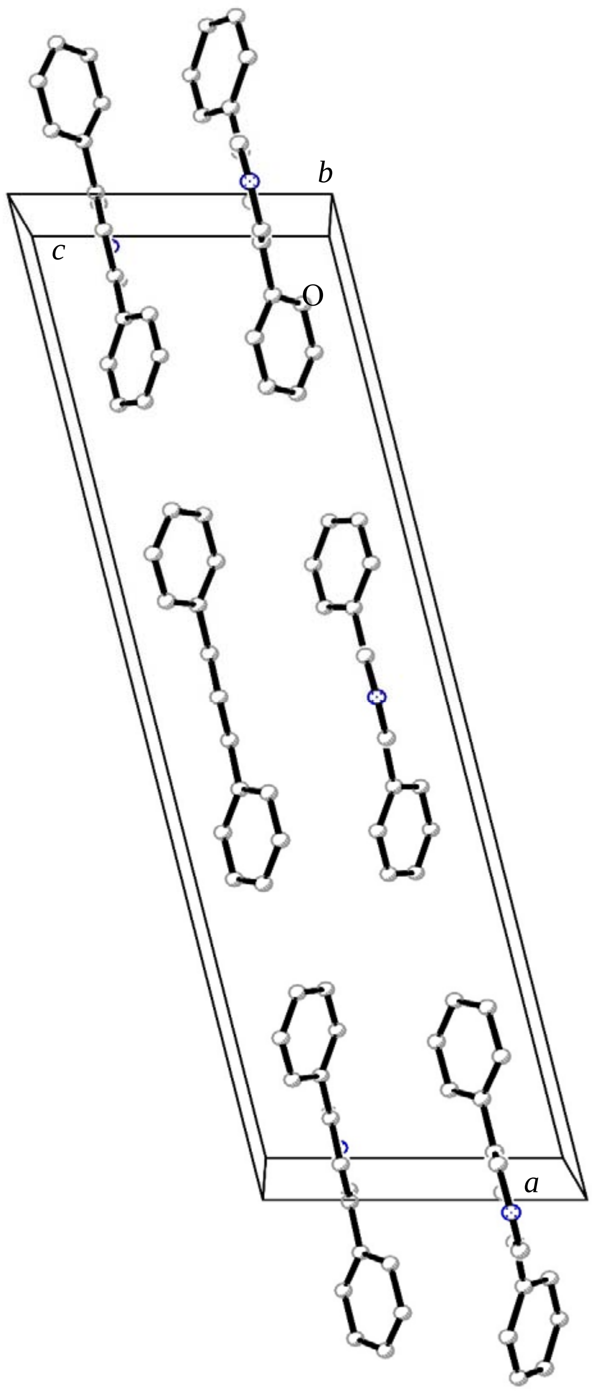

A packing view along the *b* direction

4. Part by-product's NMR spectroscopic data and GC-MS

(1) (*E*)-1,3-diphenylpropene [5]: it was isolated in 75% yield from the typical experimental procedure.

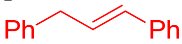

^{1}H NMR (400 MHz, $CDCl_3$): δ 7.65 – 7.38 (m, 10H), 6.74 – 6.54 (m, 2H), 3.78 – 3.76 (m, 2H); ^{13}C NMR (100 MHz, $CDCl_3$) δ 140.2, 137.5, 131.1, 129.2, 128.7, 128.5(2C), 127.1, 126.26, 126.21, 39.4; GC-MS m/z: 194 (M^+).

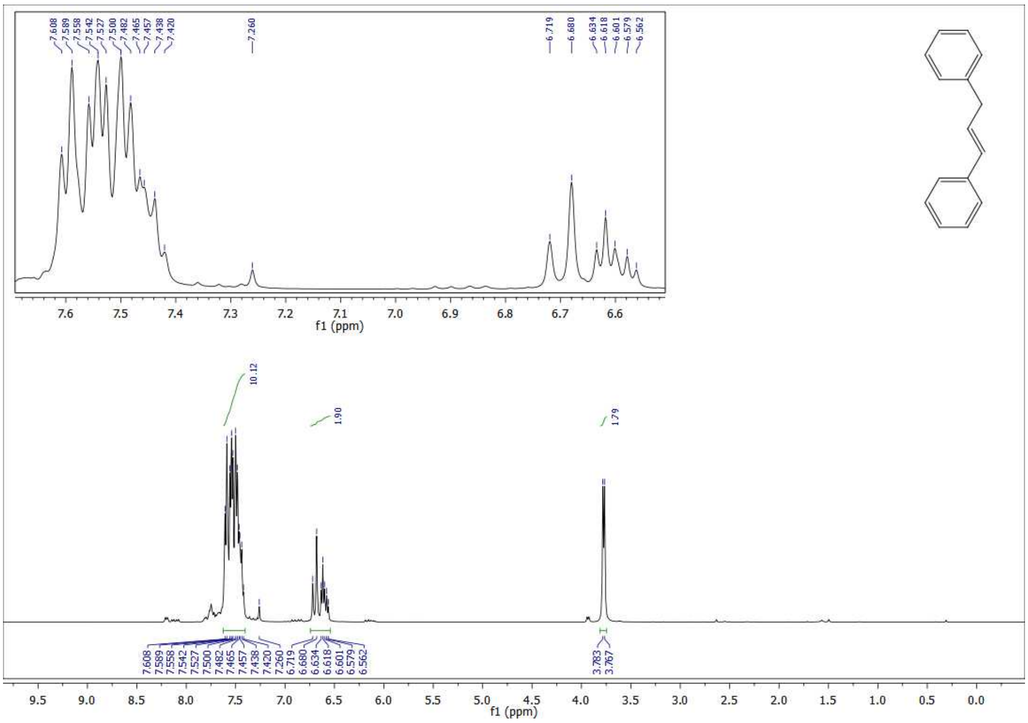

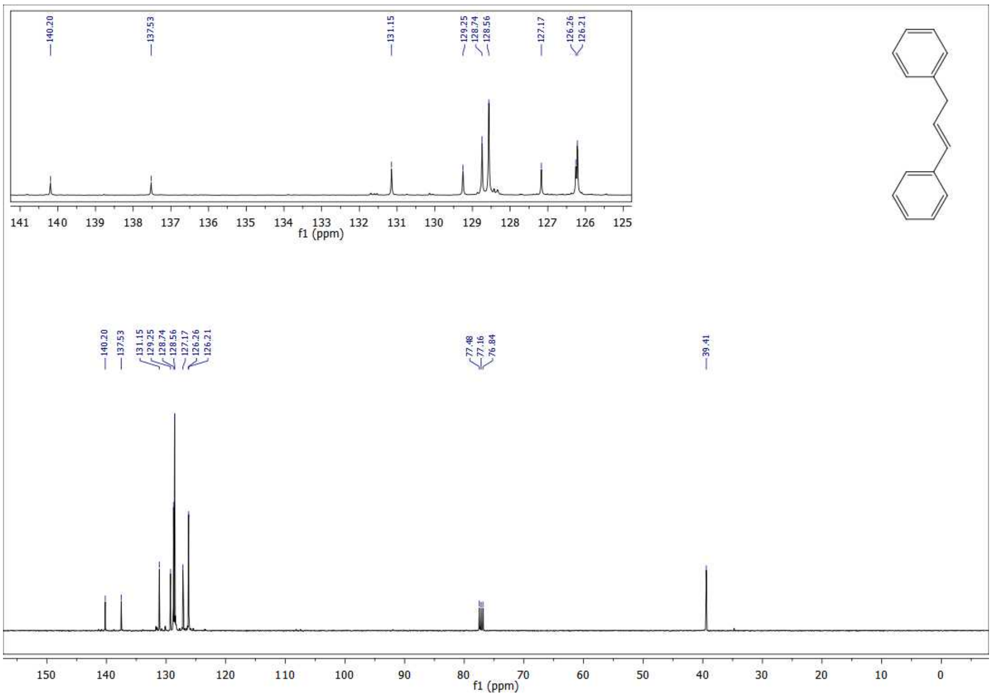

(2) (*E*)-1,3-di(4-chlorophenyl)propene [5]: 64%.

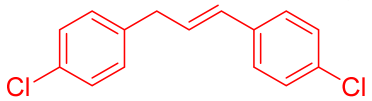

^{1}H NMR (400 MHz, $CDCl_3$) δ 7.32 – 7.23 (m, 6H), 7.19 – 7.11 (m, 2H), 6.42 – 6.22 (m, 2H), 3.50 (d, *J* = 6.4 Hz, 2H); ^{13}C NMR (100 MHz, $CDCl_3$) δ 138.3, 135.8, 132.9, 132.1, 130.3, 130.1, 129.4, 128.8, 128.7,127.4, 38.7; GC-MS m/z: 262 (M^+).

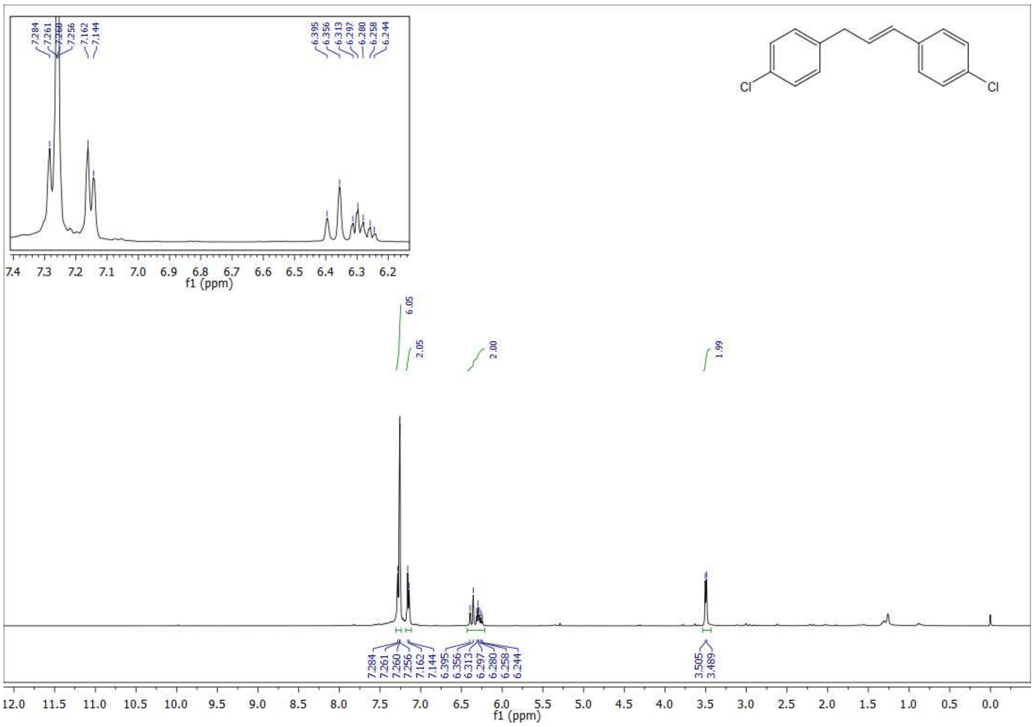

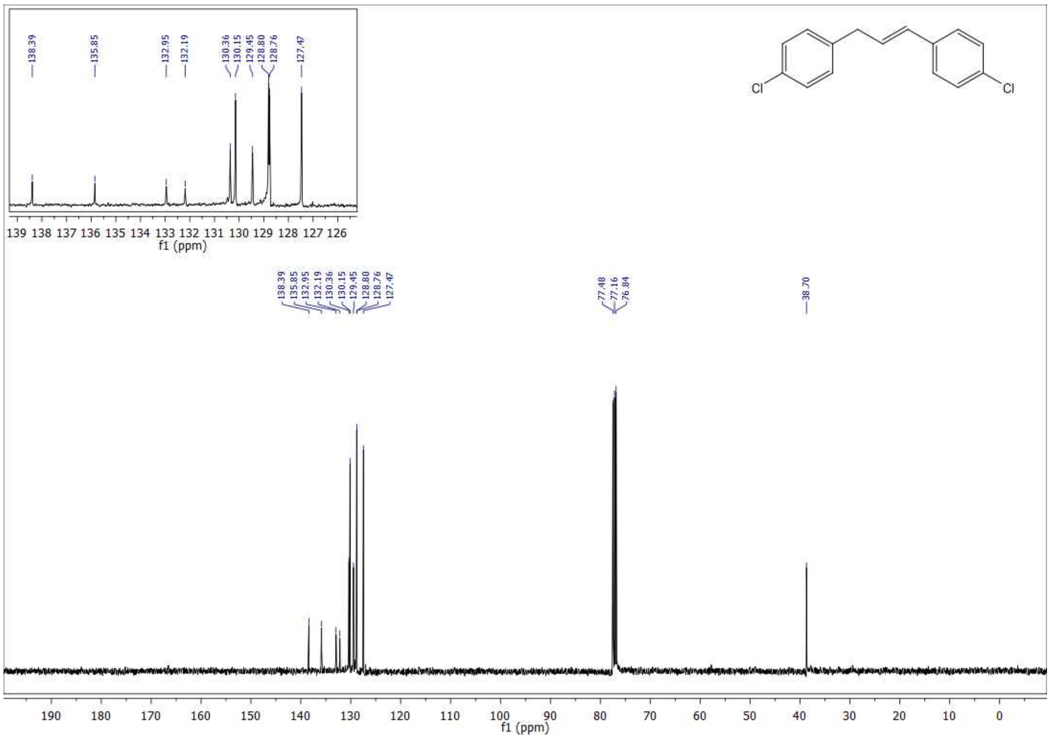

(3) (*E*)-1,3-di(3-methoxyphenyl)propene [5]: 39%.

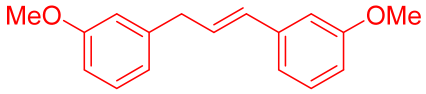

^{1}H NMR (400 MHz, $CDCl_3$) δ 7.29 – 7.19 (m, 2H), 6.98 (d, *J* = 7.7 Hz, 1H), 6.93 (s, 1H), 6.89 – 6.76 (m, 4H), 6.49 – 6.32 (m, 2H), 3.81 (s, 6H), 3.54 (d, *J* = 6.5 Hz, 2H); ^{13}C NMR (100 MHz, $CDCl_3$) δ 159.8, 141.8, 139.0, 131.1, 129.5, 129.4, 121.1, 118.9, 114.4, 112.9, 111.6, 111.4, 55.2, 39.4. GC-MS m/z: 254 (M^+).

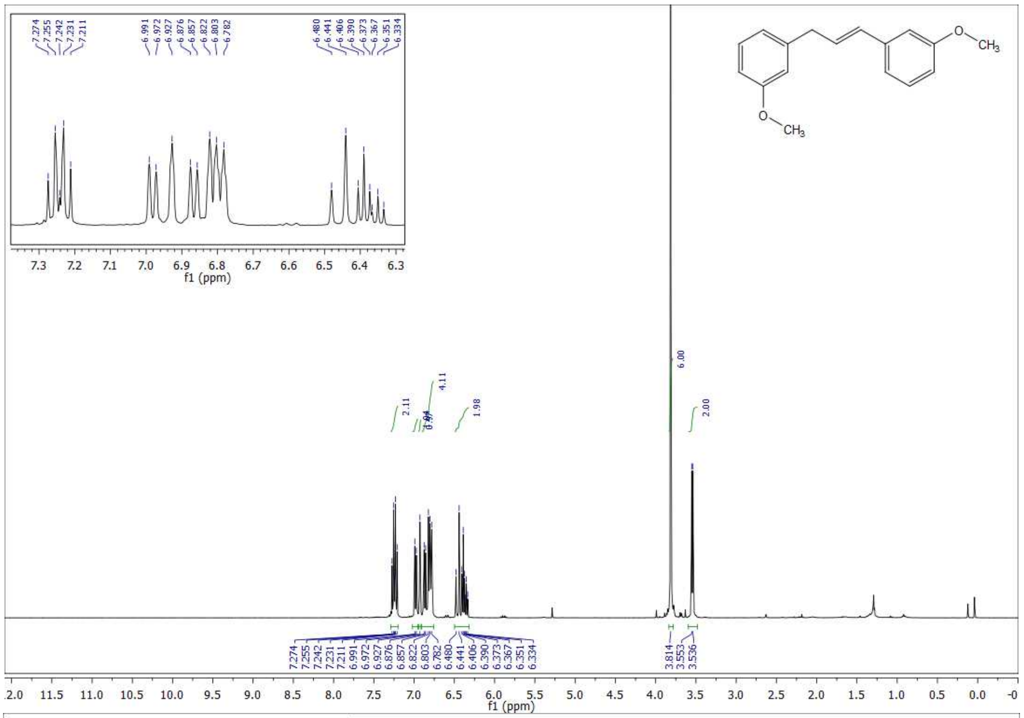

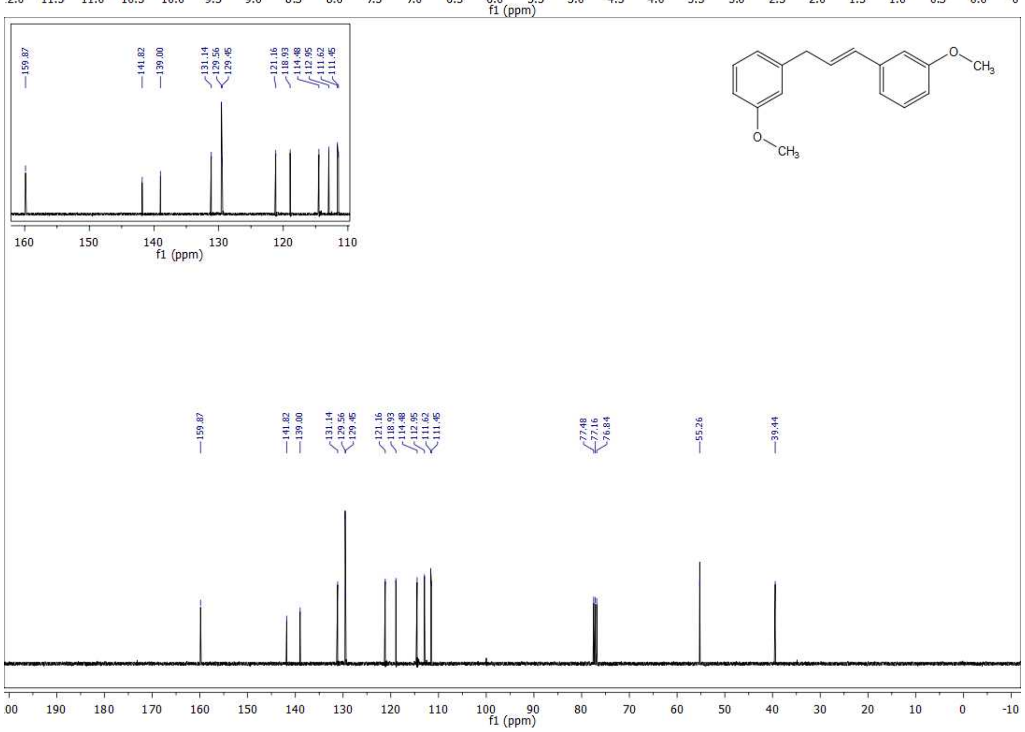

(4) (*E*)-2,2'-(Prop-1-ene-1,3-diyl)dithiophene [5]: 77%.

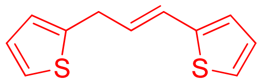

^{1}H NMR (400 MHz, $CDCl_3$) δ 7.17 – 7.11 (m, 2H), 6.99 – 6.90 (m, 3H), 6.88 – 6.84 (m, 1H), 6.62 (d, *J* = 15.6 Hz, 1H), 6.25 – 6.17 (m, 1H), 3.70 (d, *J* = 6.8 Hz, 2H); ^{13}C NMR (100 MHz, $CDCl_3$) δ 142.8, 142.3, 128.0, 127.4, 127.0, 125.2, 124.9, 124.6, 123.99, 123.92, 33.2; GC-MS m/z: 206 (M^+).

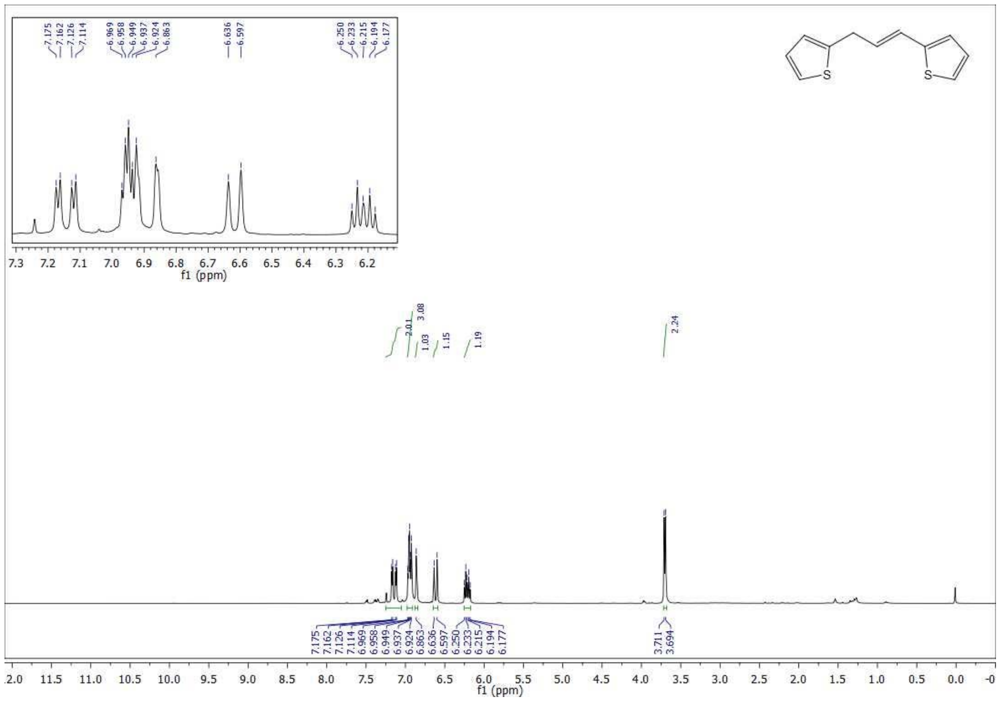

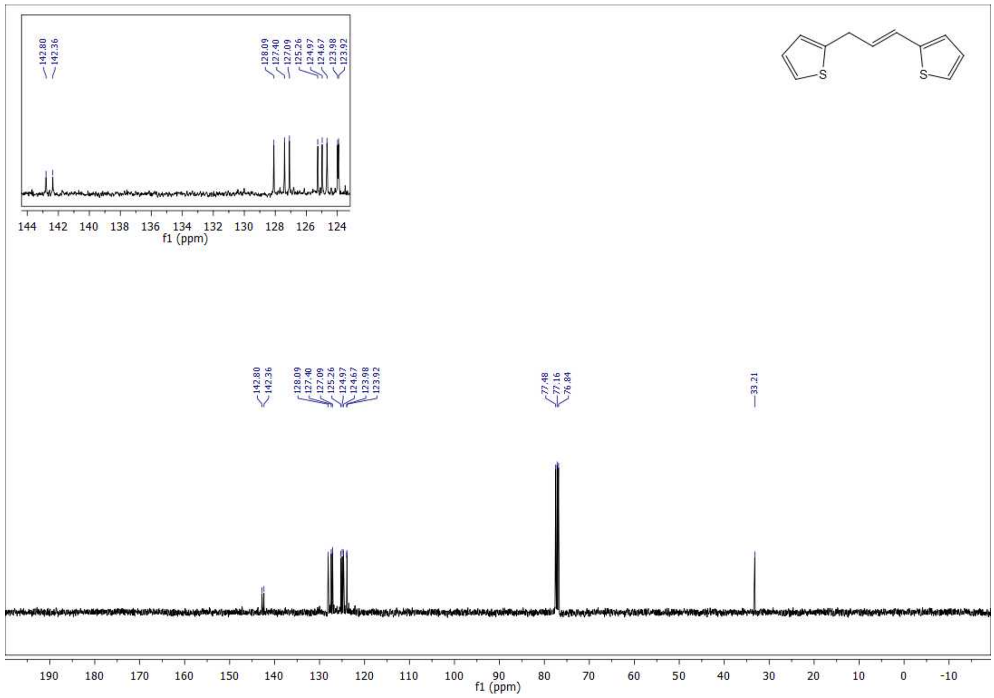

(5) GC-MS of benzoic acid

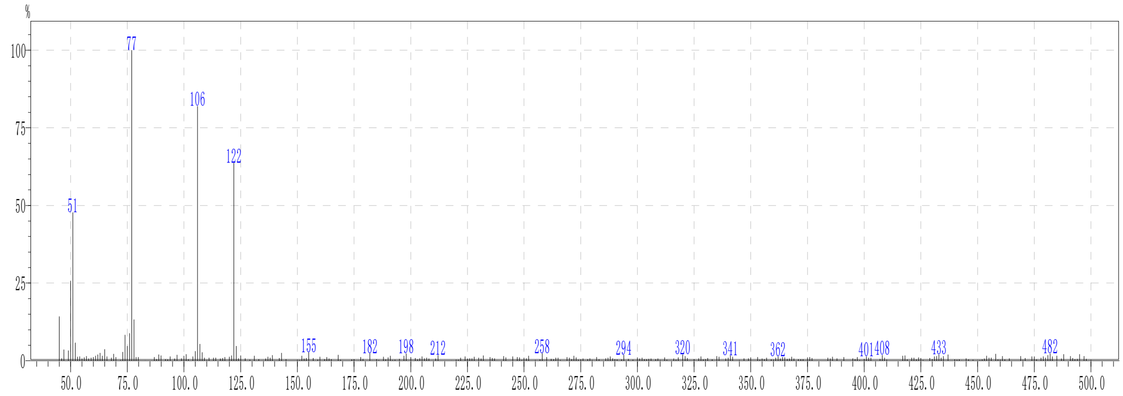

(6) GC-MS of *para*-methyl benzoic acid

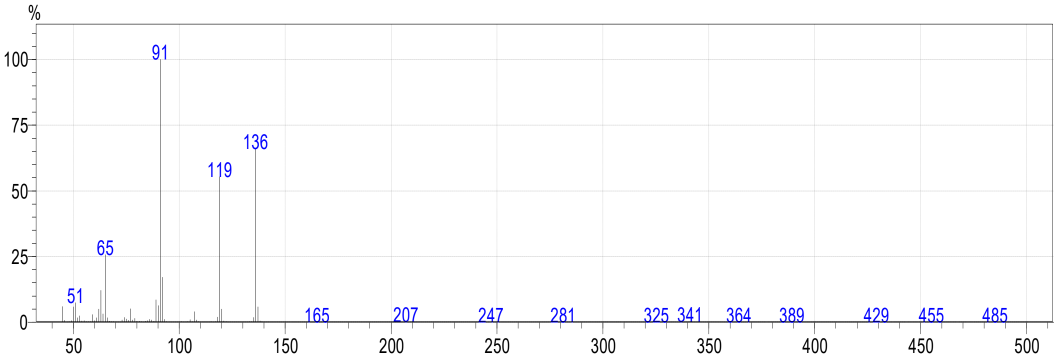

References

[5] Cao, F.; Duan, Z.-C.; Zhu, H.; Wang, D. Deoxygenative coupling of 2-aryl-ethanols catalyzed by unsymmetrical pyrazolyl-pyridinyl-triazole ruthenium. *Mol. Catal.* **2021**, *503*, 111391.
